# Valley phenomena in the candidate phase change material WSe_2(1−x)_Te_2x_

**Published:** 2020

**Authors:** Sean M. Oliver, Joshua Young, Sergiy Krylyuk, Thomas L. Reinecke, Albert V. Davydov, Patrick M. Vora

**Affiliations:** 1 Department of Physics and Astronomy, George Mason University, Fairfax, VA, USA.; 2 Quantum Materials Center, George Mason University, Fairfax, VA, USA.; 3 Department of Physics, Applied Physics and Astronomy, Binghamton University, Vestal, NY, USA.; 4 Materials Science and Engineering Division, National Institute of Standards and Technology, Gaithersburg, MD, USA.; 5 Theiss Research, La Jolla, CA, USA.; 6 Naval Research Laboratory, Washington, DC, USA.

## Abstract

Alloyed transition metal dichalcogenides provide an opportunity for coupling band engineering with valleytronic phenomena in an atomically-thin platform. However, valley properties in alloys remain largely unexplored. We investigate the valley degree of freedom in monolayer alloys of the phase change candidate material WSe_2(1−x)_Te_2x_. Low temperature Raman measurements track the alloy-induced transition from the semiconducting 1H phase of WSe_2_ to the semimetallic 1T_d_ phase of WTe_2_. We correlate these observations with density functional theory calculations and identify new Raman modes from W-Te vibrations in the 1H-phase alloy. Photoluminescence measurements show ultra-low energy emission features that highlight alloy disorder arising from the large W-Te bond lengths. Interestingly, valley polarization and coherence in alloys survive at high Te compositions and are more robust against temperature than in WSe_2_. These findings illustrate the persistence of valley properties in alloys with highly dissimilar parent compounds and suggest band engineering can be utilized for valleytronic devices.

The valley contrasting properties of monolayer semiconducting transition metal dichalcogenides (TMDs) provide the possibility of manipulating information using the valley pseudospin^1–3^ in direct analogy to spintronics^4^. Devices that employ this schema for computation, commonly referred to as valleytronics, benefit from optical or electrical manipulation of the valley index, spin–valley locking, low power consumption, and the absence of Joule heating^2^. These advantages have stimulated vigorous investigations into the properties of two-dimensional (2D) semiconducting TMDs with an emphasis on exploring the optical interband selection rules that connect photon polarization to valley index^1–3^.

The first studies of monolayer TMDs focused solely on the photoluminescence (PL) from molybdenum disulfide (MoS_2_)^5,6^; however, attention shifted to valley-dependent studies^7,8^. Soon after, monolayer tungsten diselenide (WSe_2_) was found to be a superior TMD for valleytronics, exhibiting both exciton valley polarization and valley coherence^9–15^. Valley polarization describes the probability of an exciton created in a valley to remain there until recombination, while valley coherence quantifies the probability of an exciton to remain in a superposition state of K and K′ valleys before recombining. At low temperature, the neutral exciton (X^0^) in bare WSe_2_ monolayers exhibits a valley polarization ranging from 40% to 70% depending on the laser energy^9,11,16^. The depolarization of valley excitons is believed to be governed by a combination of intervalley electron–hole exchange interactions^8,17^, phonon-assisted intervalley scattering^7,18^, and Coulomb screening of the exchange interaction^19,20^. In addition, WSe_2_ holds the record for the largest degree of X^0^ valley coherence in bare monolayers (≈40% at cryogenic temperatures)^9^.

Alloying can increase the technological potential of valleytronic TMDs by combining valley contrasting properties with band engineering^21^. Furthermore, alloys of structurally distinct TMDs may also enable phase change memory technologies^22–24^ by reducing the energy barrier between semiconducting and semimetallic states^25^. Few studies, however, have explored valley phenomena in alloys despite the heavy interest in this field. The first exploration of valley properties in alloys focused on Mo_1−*x*_W_*x*_Se_2_ at 5 K and found that there was a transition from the intrinsic valley polarization of MoSe_2_ to WSe_2_ as the transition metal content was varied^26^. Experiments on a WS_0.6_Se_1.4_ alloy demonstrated a valley polarization of ≈31% at 14 K, which was much lower than that of both WS_2_ and WSe_2_^27^. A recent study of WS_*x*_Te_2−*x*_ found that the room temperature valley polarization increased from 3% in WS_2_ to 37% in an unspecified alloy composition^28^. The limited information regarding valley polarization in alloyed TMDs is a serious oversight as future valleytronic technologies will likely rely heavily on band engineering.

In this study, we examine the low-temperature optical properties of monolayer WSe_2(1−*x*)_Te_2*x*_, where the endpoints are the valleytronic semiconductor 1H-WSe_2_ and the topological semimetal 1T_d_-WTe_2_. Prior studies of this alloy system were performed at 300 K and focused on unpolarized optical measurements^29^ as well as electronic transport characterization^30^. Our study focuses on the impact of alloying on the valley excitons of WSe_2(1−*x*)_Te_2*x*_ monolayers encapsulated in hexagonal boron nitride (hBN). We ensure good interfacial contact in the heterostructure by cleaning with the nano-squeegee method^31^, which substantially improves the valley polarization. Raman measurements at 5 K show that increasing Te composition leads to the shifting and splitting of vibrational modes as well as the appearance of new modes unique to alloys. Polarization-resolved Raman measurements coupled with density functional theory (DFT) calculations of the theoretical 1H-WTe_2_ phase allow us to assign these alloy-only features as resulting from W-Te vibrations in the 1H WSe_2(1−*x*)_Te_2*x*_ structure. Temperature-dependent, polarization-resolved PL measurements of the 1H-phase alloys demonstrate band gap tunability alongside the presence of new ultra-low energy emission features. We suggest that these features are from deep mid-gap states that originate from the large difference in the WSe and W-Te bond lengths. Despite the presence of structural disorder in the lattice, valley polarization is found to survive the alloying process for *x* ≤ 0.14 while valley coherence is present for alloys *x* ≤ 0.37, and interestingly, we demonstrate that alloys have the ability to sustain these valley properties at higher temperatures than pure WSe_2_. These findings illustrate the persistence of valley phenomena in significantly disordered alloys and point the way toward optimization of TMDs for novel phase change memories that naturally integrate with valleytronic devices.

## Results

### WSe_2(1−x)_Te_2x_ structure and vibrational modes.

**Fig. 1:**
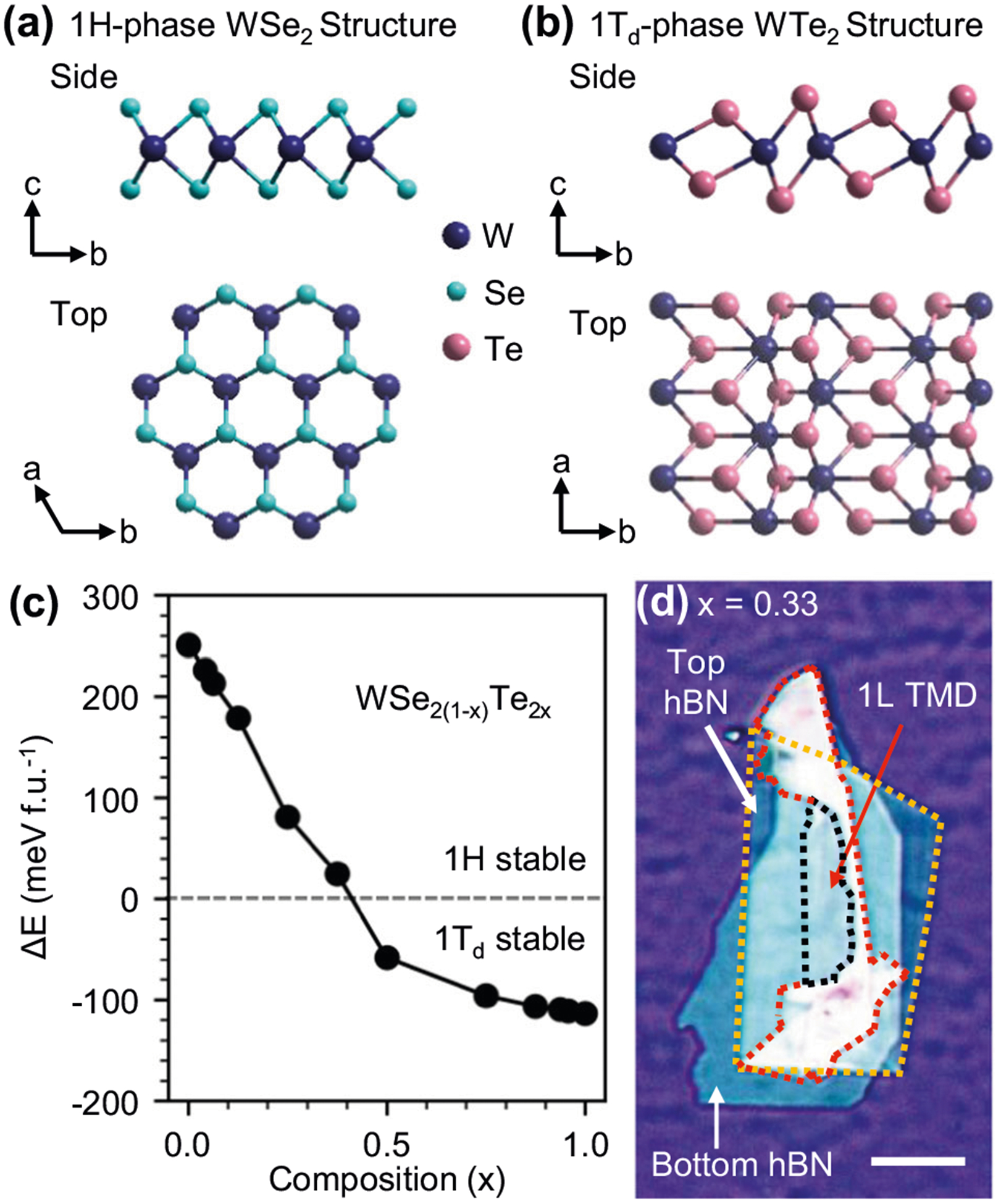
Structural phases and van der Waals heterostructures of WSe_2(1−x)_ Te_2x_. Side and top view of **a** monolayer 1H-WSe_2_ and **b** monolayer 1T_d_-WTe_2_. **c** Composition-dependent phase diagram determined from density functional theory calculations indicating a phase boundary at *x* = 0.4. **d** Optical image of a hexagonal boron nitride (hBN)-encapsulated *x* = 0.33 monolayer deposited onto a SiO_2_/Si substrate. The transition metal dichalcogenide (TMD) alloy is outlined in red and the monolayer (1L) portion of that flake is outlined in black. The top layer of hBN is outlined in yellow. The white scale bar is 20 μm.

Interpretation of our experimental data is guided by DFT modeling of the WSe_2(1−*x*)_Te_2*x*_ phase diagram. The crystal structure of the endpoint compounds 1H-WSe_2_ (*x* = 0) and 1T_d_-WTe_2_ (*x* = 1) are shown in Fig. 1a, b. Regarding crystal structure notation, we refer to T_d_ monolayers as being in the 1T_d_ phase. Previously, T_d_ and 1T’ notations have been used interchangeably for T_d_ monolayers since the only difference between these structures in the bulk was believed to be a slight shift of layers relative to one another. Since the T_d_ structure contains three layers in its unit cell while the 1T’ structure contains one layer, it was assumed that monolayers isolated from a bulk T_d_ crystal were in the 1T’ phase. We make the 1T_d_ notation distinction in light of a recent report which has indicated, unlike the 1T’ phase, that inversion symmetry is broken in the monolayer T_d_ lattice^32^. We find from our DFT calculations, in agreement with previous studies^29,30^, that the 1H phase is stable with a Te concentration of *x* ≤ 0.4, which we illustrate in Fig. 1c. For *x* ≥ 0.5, 1T_d_ becomes the lowest energy phase. As a result, we expect valleytronic optical properties for the 1H alloys corresponding to *x* ≤ 0.4 and semimetallic behaviors for the 1T_d_ alloys *x* ≥ 0.5. These predictions are consistent with our Raman and PL measurements described in detail below.

**Fig. 2:**
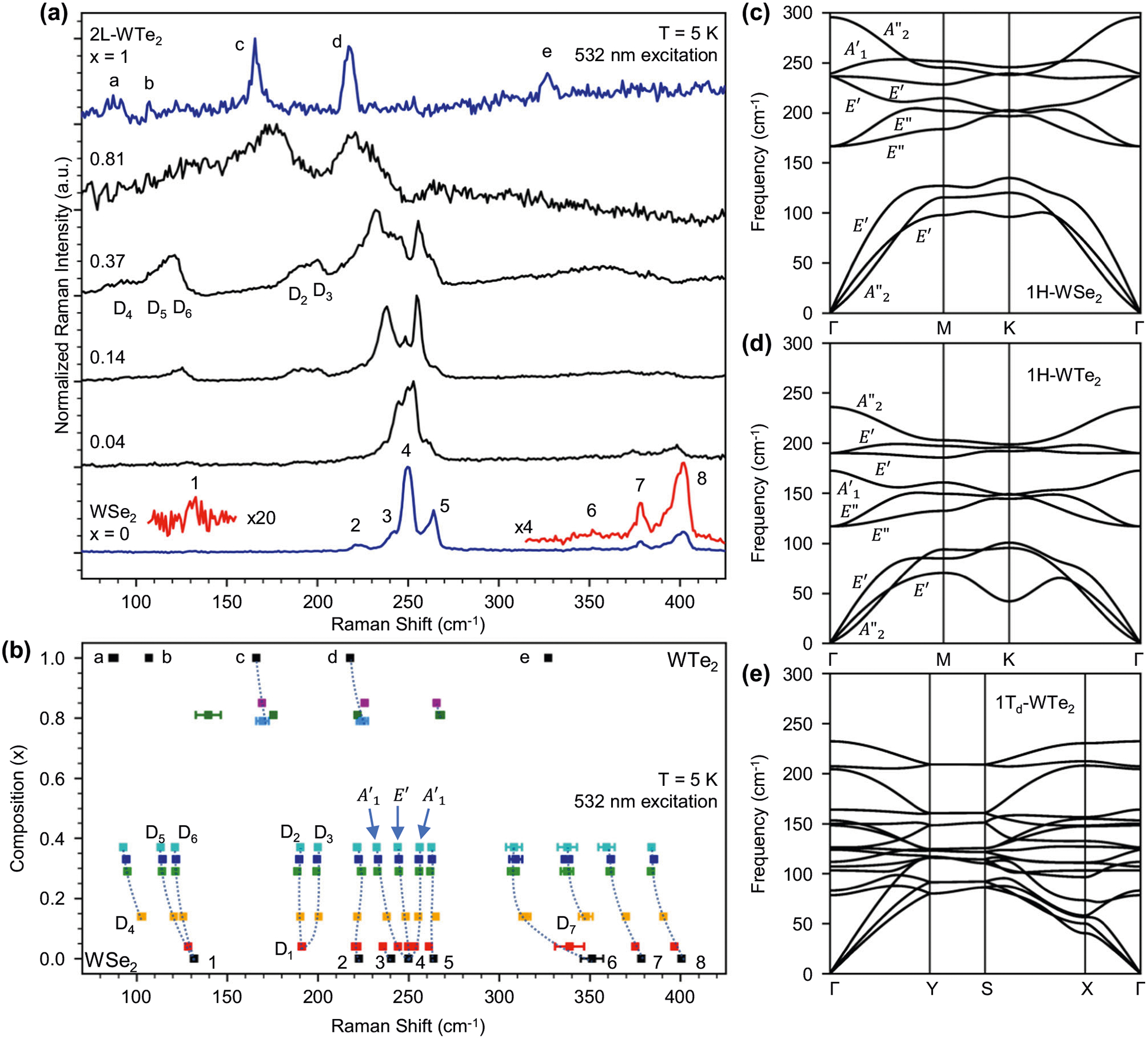
Composition-dependent Raman spectra of WSe_2(1−x)_Te_2x_ and phonon band structures of WSe_2_ and WTe_2_. **a** Raman measurements of monolayer WSe_2_, bilayer (2L) WTe_2_, and select monolayer WSe_2(1−*x*)_Te_2*x*_ alloys taken at 5 K with 532 nm excitation. Vibrational modes in WSe_2_ and WTe_2_ are identified with numbers and letters, respectively, and their assignments can be found in Table 1. Parts of the WSe_2_ spectra are scaled for clarity. **b** Peak positions extracted from Raman measurements at 5 K for different alloy compositions *x*. Peaks identified in **a** for WSe_2_ and WTe_2_ are labeled with their respective numbers and letters. New alloy-induced vibrational modes are labeled *D*_i_ (i = 1, 2, 3, …). The composition-dependent shifting and the splitting of peaks are tracked with dotted lines. The error bars in **b** are one standard deviation and in most cases are smaller than the data point. c–e Phonon band structures calculated for 1H-WSe_2_, 1H-WTe_2_, and 1T_d_-WTe_2_.

Prior to optical measurements, monolayers are encapsulated in an hBN heterostructure, as shown in Fig. 1d, to protect from the degradative effects due to exposure to the atmosphere and to provide a uniform dielectric environment^33^. We explore the effects of alloying on crystal structure through low-temperature Raman spectroscopy of hBN-encapsulated monolayer WSe_2(1−*x*)_Te_2*x*_, as shown in Fig. 2a. This Raman data is fit with multiple Lorentzian peaks to extract mode frequencies, which are plotted in Fig. 2b with alloy composition *x*. Examples of Lorentzian fits to Raman data for select alloys can be seen in Supplementary Fig. 1.

It is instructive to first examine the properties of the endpoints WSe_2_ (*x* = 0) and WTe_2_ (*x* = 1) that appear in blue in Fig. 2a. WSe_2_, which naturally crystallizes in the 1H phase, is in the *D*_3h_ point group and exhibits modes with $A_{1}^{\prime }$ and E′ symmetries. In agreement with prior observations^34,35^, we identify eight Raman peaks in WSe_2_ at 132, 223, 240, 250, 264, 351, 378, and 401 cm^−1^. These modes and their symmetries are summarized in Table 1. The dominant feature in the WSe_2_ Raman spectra at 250 cm^−1^ (labeled peak 4 in Fig. 2a, b) is an $A_{1}^{\prime }$ symmetry mode where the transition metal is fixed and the chalcogens vibrate perpendicular to the basal plane^36,37^. In monolayer WSe_2_, this mode overlaps with an *E*′ symmetry mode where the chalcogen and transition metal layers both vibrate in plane but out of phase^36,37^. These findings agree with prior calculations^34,36,37^ as well as our own DFT modeling of the WSe_2_ phonon band structure that predicts the 0 K frequency of the dominant $A_{1}^{\prime }$ mode at 239 cm^−1^ and the *E*′ mode at 236 cm^−1^ (Fig. 2c). Additional monolayer WSe_2_ modes are enhanced at 5 K, which are usually assigned as disorder-activated finite momentum phonons, combination modes, or difference modes^34,35,38^. We find that previous assignments of these features to Raman difference modes conflict with our low-temperature data. Difference modes originate from a two-phonon process where one phonon is absorbed (anti-Stokes process) and another is created (Stokes process). Since this requires the presence of a phonon before photoexcitation, its occurrence is expected to follow a Boltzmann-like temperature dependence that disappears at cryogenic temperatures^39^. The presence of these so-called difference modes in our measurements at 5 K calls this assignment into question. Thus it is more likely that these features are combination modes for which there are many possible assignments (Table 1); however, there is presently no explanation for the mode at 132 cm^−1^.

We now discuss the Raman spectrum of WTe_2_, which is presented in Fig. 2a. Our attempts to exfoliate large-area monolayers of WTe_2_ were met with limited success, which may be due to the rapid oxidation rate of this material^40^. In contrast, we found it straightforward to achieve large-area bilayer WTe_2_, which is known to be less susceptible to oxidation^40^. The Raman spectra of bilayer and monolayer WTe_2_ are nearly identical^41^, which lets us safely use bilayer WTe_2_ to discriminate between 1T_d_- and 1H-WSe_2(1−*x*)_ Te_2*x*_. Bilayer WTe_2_ (T_d_ phase) belongs to the C_2v_ point group, and so only *A*_1_ and *A*_2_ symmetry modes can be observed^42^. Five peaks in the 70–425 cm^−1^ range are present at 87, 107, 166, 218, and 327 cm^−1^ and are labeled with letters in Fig. 2a, b. Assignment of the mode symmetries is based on prior studies of WTe_2_^41,43^ and is included in Table 1. The feature at 327 cm^−1^, labeled peak e, has not been previously observed, and we assign it as either a second-order overtone of the 166 cm^−1^ A_1_ mode (peak c) or a combination of the 107 cm^−1^ A_2_ and 218 cm^−1^ A_1_ modes (peaks b and d, respectively).

Low-temperature Raman measurements of the alloys (black curves of Fig. 2a) reveal fascinating new details regarding the vibrational modes that were not observed in a previous study of WSe_2(1−*x*)_Te_2*x*_ alloys owing to thermal broadening at 300 K (see Supplementary Fig. 2 for comparison of 5 and 300 K spectra). Alloys with *x* ≤ 0.37 are in the 1H phase, which is further supported by the presence of PL in these samples to be discussed later, and exhibit complex mode structures in the ≈230–275 cm^−1^ range. Polarization-resolved Raman measurements (Supplementary Fig. 3) reveal that the dominant WSe_2_ $A_{1}^{\prime }$ mode at 250 cm^−1^ splits into two peaks at 244 and 253 cm^−1^ for *x* = 0.04 (see red points in Fig. 2b). This differs from studies of WS_2(1−*x*)_Se_2*x*_ where this primary out-of-plane WSe_2_ feature typically only shifts with alloying^44,45^. The splitting of out-of-plane vibrational modes with alloying, however, has been seen in MoS_*x*_Se_2−*x*_ monolayers^46,47^ and has been carefully documented in few-layer MoS_*x*_Se_2−*x*_^48^. Jadczak et al. attributed the splitting of this feature in alloys to the polarization of the alloy unit cell due to the substitution of heavier chalcogens that introduce different force constants in the lattice^48^. Thus, owing to the splitting of the primary $A_{1}^{\prime }$ mode, we find that the *E*′ mode is distinguishable from the $A_{1}^{\prime }$ mode in alloy monolayers and only slightly shifts to lower frequencies with increasing *x*.

Several other WSe_2_-like vibrational modes show sensitivity to alloying for 1H-phase compositions *x* ≤ 0.37. Second-order finite momentum peaks 6, 7, and 8 of Fig. 2a, b shift to lower frequencies and broaden with increasing alloy composition. This alloy data may clarify disagreements in the literature on whether $A_{1}^{\prime }$ or *E*^′^ phonons are the dominant contributor to these higher-order modes^34,35^. The band predictions of 1H-WSe_2_ in Fig. 2c show both branches of the *E*^′^ mode that originate at 236 cm^−1^ shift to lower frequencies away from the *Γ* point. This behavior is opposite to that of the $A_{1}^{\prime }$ mode, which originates at 239 cm^−1^ and shifts to higher frequencies away from the Γ point. Since peaks 6, 7, and 8 broaden asymmetrically on the lower frequency side of their centers as *x* is increased, this may indicate that the *E*^′^ mode, rather than the $A_{1}^{\prime }$ mode, contributes to these higher-order processes. The shifting and broadening of the WSe_2_ modes for compositions *x* ≤ 0.37 indicate that alloying introduces significant disorder into the lattice but globally maintains the 1H phase.

A particularly interesting alloy-induced feature resolved in 1H samples is the Raman peak labeled D_1_ at 191 cm^−1^ in *x* = 0.04 (red points in Fig. 2b). This feature splits into the two peaks labeled D_2_ and D_3_ at 190 and 200 cm^−1^, respectively, as *x* is increased. Polarization-resolved Raman measurements (Supplementary Fig. 3) indicate that these features have $A_{1}^{\prime }$ symmetry and DFT calculations of the 1H-WSe_2_ phonon band structure (Fig. 2c) show no $A_{1}^{\prime }$ phonon modes present in this range. We therefore calculate the phonon band structure of metastable 1H-WTe_2_ phase in Fig. 2d, which predicts an $A_{1}^{\prime }$ *Γ*-point mode at 172 cm^−1^. This mode is the closest to the observed results and so we assign the D_1_ peak to an $A_{1}^{\prime }$ mode arising from W-Te vibrations in the 1H WSe_2(1−*x*)_Te_2*x*_ alloys. We attribute its splitting to increasing force constant variations introduced into the lattice with noticeable Te content as discussed previously for the primary $A_{1}^{\prime }$ mode of WSe_2_^48^. Raman measurements reveal several other new peaks in the 1H alloys, which we label D_4_, D_5_, D_6_, and D_7_ in Fig. 2b. These polarization-independent features (see Supplementary Fig. 3) most likely arise from either finite momentum WSe_2_-like phonons or WSe_2_ combination modes. We exclude difference modes based on the use of low-temperature spectroscopy as discussed previously. Possible assignments are *E*^′^ M for D_6_ and *E*^′^ + *E*^′^(*M*) for D_7_, while D_4_ and D_5_ remain unassigned but may originate from combinations of 1H-WSe_2_ and 1H-WTe_2_ modes. As WSe_2(1−*x*)_Te_2*x*_ transitions to the 1T_d_ phase with alloying, the Raman spectra for *x* ≥ 0.79 show the two primary *A*_1_ vibrational modes of pure WTe_2_, labeled peaks c and d in Fig. 2a, b. These features are shifted and broadened due to alloy disorder in agreement with other WTe alloys^49^. The phonon band structure of 1T_d_-WTe_2_ is shown in Fig. 2e for comparison with that of 1H-WTe_2_ in Fig. 2d.

### Excitonic properties.

**Fig. 3:**
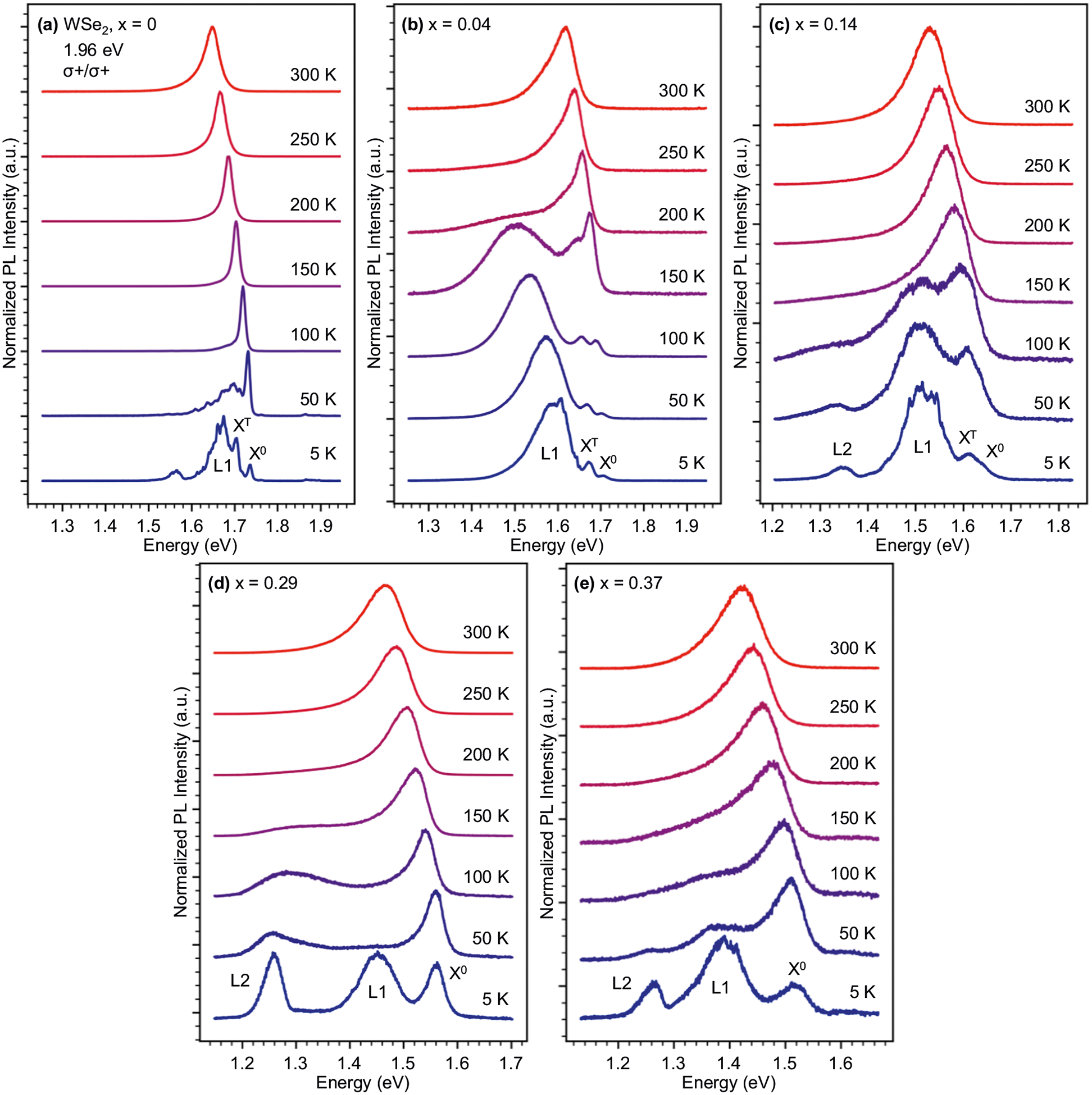
Temperature-dependent photoluminescence (PL) of 1H-WSe_2(1−x)_Te_2x_. PL measurements (1.96 eV excitation) of **a** WSe_2_, as well as 1H-phase WSe_2(1−*x*)_Te_2*x*_ alloys corresponding to **b** *x* = 0.04, **c** *x* = 0.14, **d** *x* = 0.29, and **e** *x* = 0.37. Excitation and collection are done with right circularly polarized light (*σ*+). The neutral exciton (X^0^) and trion (X^T^) are labeled where appropriate. Emission at 300 K is dominated by X^0^, which has a low-energy tail resulting from the presence of X^T^. As the temperature is decreased, X^0^ and X^T^ sharpen and blueshift while the localized exciton features L1 and L2 begin to dominate the spectra.

In Fig. 3a–e, we present temperature-dependent PL spectra for representative 1H-phase alloys. Each spectrum has been normalized by the maximum intensity and was excited with right circularly polarized light (*σ*+) at 1.96 eV. The collected PL is passed through a waveplate/analyzer combination to select *σ*+ emission. At 300 K, the PL spectra show contributions from both the neutral exciton (X^0^) and the trion (X^T^)^12^. Increasing the Te concentration causes both features to redshift due to the lower band gap of 1H-WTe_2_^50^. As temperature decreases from 300 to 5 K, X^0^ and X^T^ sharpen, blueshift, and weaken in intensity. The latter behavior is due to the sign of the conduction band spin–orbit coupling, which makes the lowest exciton state dark^51^. The sign of the spin–orbit coupling is the same for all W-based TMDs^51^ and so we expect no change in the optical activity of the lowest exciton state with Te substitution. The presence of broad features at lower energies compared to X^0^ and X^T^ originate from a combination of higher-order excitonic complexes^52^ and localized exciton states from lattice defects, strain, and residual impurities introduced during fabrication^9,12,53^.

**Fig. 4:**
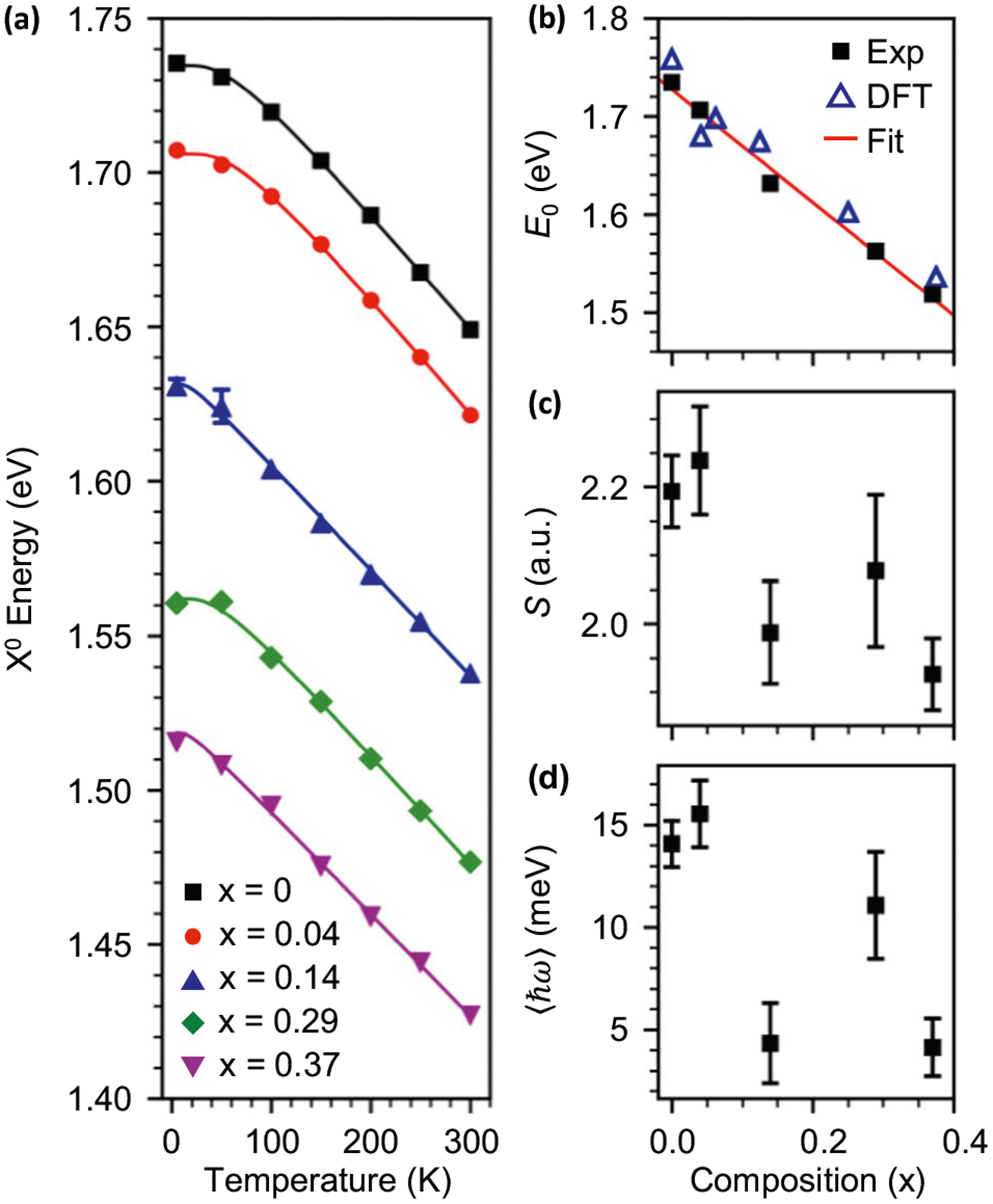
Composition- and temperature-dependent exciton energies. **a** Neutral exciton (X^0^) energies extracted from temperature-dependent photoluminescence measurements (1.96 eV excitation). Excitation and collection are done with right circularly polarized light. The solid lines are fits to Eq. (1). The compositional dependence of the extracted parameters *E*_0_, *S*, and *ħω* are plotted in **b–d**, respectively. *E*_0_ is found to be tunable with alloying, while *S* and *ħω* are found to decrease with increasing alloy composition *x*. In **b**, we plot density functional theory (DFT)-predicted optical band gaps as blue triangles, while the red curve is a fit to a line used to extract a 0-K band gap for 1H-WTe_2_ of 1.15 eV. Error bars shown in all panels are equal to one standard deviation.

We explore band gap tunability of 1H-WSe_2(1−*x*)_Te_2*x*_ by extracting X^0^ energy as a function of temperature, which is plotted in Fig. 4a. This data is fit with a semi-empirical formula for temperature-dependent optical band gaps given by^54^

(1) $$E_{\text{g}}\left(T\right)\;=\;E_{0}\;-\;S\langle \hbar \omega \rangle \left[\text{coth}\left(\frac{\langle \hbar \omega \rangle }{2kT}\right)\;-\;1\right]$$

where *E*_0_ is the gap at absolute zero, *S* is a dimensionless electron–phonon coupling parameter, *ħω* is an average phonon energy, and *k* is the Boltzmann constant. This formula models the reduction in band gap with increasing temperature due to a combination of increasing lattice constants and exciton–phonon coupling^54^. Fits of Eq. (1) to our data are presented as solid lines in Fig. 4a and the compositional dependence of *E*_0_, *S*, and *ħω* from these fits are plotted in Fig. 4b–d. Figure 4b shows that *E*_0_(i.e., X^0^ energy) varies in 1H-WSe_2(1−*x*)_Te_2*x*_ from ≈1.735 eV in WSe_2_ to ≈1.519 eV in the *x* = 0.37 alloy. These experimental results agree extremely well with the optical band gaps of the alloys computed using DFT with the HSE06 exchange-correlation functional^55,56^, which predict a gap of 1.75 eV in 1H-phase WSe_2_ that decreases to 1.53 eV at a Te concentration of *x* = 0.375 (blue triangles of Fig. 4b). At higher *x*, the system transforms to the 1T_d_ phase (Supplementary Fig. 4), which is a semimetal. For a comparison of DFT results for the optical band gaps of all WSe_2(1−*x*)_Te_2*x*_ monolayers calculated using both the HSE06 and Perdew–Burke–Ernzerhof (PBE) functionals and the density of states determined using the HSE06 functional, see Supplementary Fig. 4. Since the alloys are in the 1H phase only up to *x* = 0.37 and 1H-WTe_2_ does not exist in nature, we are unable to reliably determine the bowing parameter for *E*_0_^21^. Therefore, we instead fit *E*_0_ from *x* = 0 to 1 with a linear function as shown by the red line in Fig. 4b. From a linear extrapolation to *x* = 1, we determine the 0 K optical band gap of 1H-WTe_2_ to be 1.15 eV. Lastly, extracted values for *S* range from 1.93 to 2.24 and for *ħω* between 4 and 16 meV (32.3–129 cm^−1^) and are plotted versus *x* in Fig. 4c, d.

Elaborating on the 5 K PL spectra of Fig. 3, we find that X^T^ appears ≈30 meV below X^0^ in our data and shifts in lockstep with the neutral exciton so that there is only weak dependence of the X^0^–X^T^ binding energy on *x* (Supplementary Fig. 5). Spatial PL mapping of an *x* = 0.33 alloy sample at 5 K indicates little variation of the intensities, energies, and line widths of X^0^ and X^T^. These maps are discussed in detail in Supplementary Note 1 and Supplementary Fig. 6. In addition, we find evidence for excitonic transitions and exciton–phonon complexes at energies above X^0^ known to originate from coupling between hBN and WSe_2_ in van der Waals heterostructures^57,58^. A detailed discussion of these features is presented in Supplementary Note 2 and Supplementary Fig. 7. In 5 and 300 K reflectance measurements presented in Supplementary Fig. 8, both the A and B excitons are clearly visible and the valence band spin–orbit coupling is found to increase with Te incorporation.

Localized excitons are common in W-based TMDs owing to the long lifetime of the dark exciton ground states. We identify such features in all alloys and the parent compound WSe_2_, beginning with an emission band ≈100 meV below X^0^ hereafter referred to as L1. For all *x* > 0.04, L1 is accompanied by a second localized emission feature (L2) approximately ≈300 meV below X^0^ (Fig. 3c–e). This feature has never been observed in TMDs, alloyed or otherwise. L1 and L2 maintain a similar energy separation with respect to X^0^ at all nonzero *x* but do exhibit variations in their temperature-dependent behaviors.

The new defect band L2 must originate from a disorder unique to Te-rich alloys and may be connected to the preference of WTe_2_ to crystallize in a 1T_d_ structure. Scanning tunneling electron microscopy measurements and molecular dynamics (MD) simulations of a similar alloy system, WS_2−*x*_Te_*x*_^59^, indicate that Te substitution at levels approaching 15% can substantially modify the structure of a 1H-phase alloy. The bond lengths and lattice constants of 1H-WSe_2_ with 1H-WTe_2_ are predicted to differ by ≈7–8%^59^, which also leads to a mismatch between the metal–chalcogen bond angles. MD simulations presented by Tang et al.^59^ illustrate that these internal strains can lead to the displacement of Te atoms from the expected chalcogen site for concentrations as low as 8%. These shifts lead to the compression and stretching of neighboring hexagonal rings and in turn result in the displacement of the native chalcogen and even the W atoms. For the WS_2_–WTe_2_ alloys, continuing to increase the Te doping to 15%, 20%, and finally 25% increased the displacements of W, S, and Te atoms and ultimately drove the W atoms closer together in a prelude to the 1H–1T transition^59^. These Te concentrations compare favorably with the values at which we observe the presence of the L2 feature (Fig. 3). The presence of such substantial atomic displacements and internal strains seems to be unique to TMD alloys that are mixtures between different structural phases. We therefore suggest that the internal strains driven by Te incorporation in 1H-phase alloys create a new band of defect states (i.e., the L2 feature) lower than those typically expected from chalcogen or transition metal vacancies (i.e., the L1 feature)^53^.

### Valley phenomena.

**Fig. 5:**
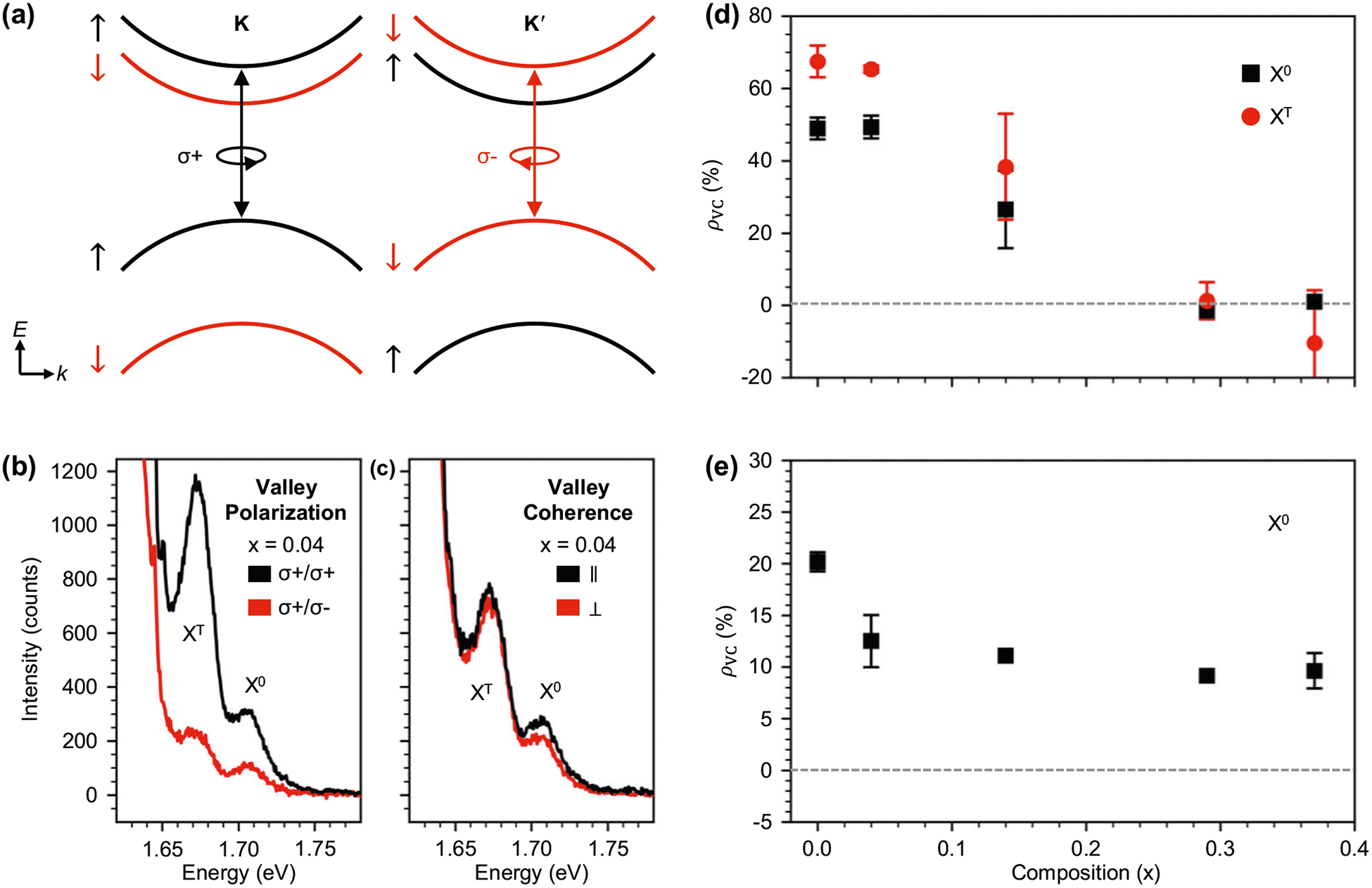
Valley properties of WSe_2(1−x)_Te_2x_ at 5 K. **a** Simplified image of the electron bands near the K and K′ points of the hexagonal Brillouin zone in monolayer WSe_2_. Valley-dependent optical selection rules couple transitions at the K (K′) valleys with *σ*+ (*σ*−) circularly polarized light. **b**, **c** show example spectra of valley polarization and valley coherence measurements for *x* = 0.04, respectively. **d** The degree of valley polarization (*ρ*_VP_) of the neutral exciton (X^0^, black squares) and the trion (X^T^, red circles) and **e** the degree of valley coherence (*ρ*_VC_) of X^0^ plotted against alloy composition *x*. The error bars shown in **d**, **e** are equal to one standard deviation.

Next, we explore valley contrasting in 1H-WSe_2(1−*x*)_Te_2*x*_ alloys at 5 K using 1.96 eV excitation with *σ*+ polarization. The schematic band structure in Fig. 5a illustrates the spin–valley polarized selection rules in WSe_2_, where transitions between the valence band and the second highest conduction band are *σ*+ (*σ*−) polarized at the K (K′) point. Any emission in the opposite polarization channel is a sign of intervalley scattering. Valley polarization is determined by measuring co- and cross-polarized PL spectra in the circular basis as shown in Fig. 5b. The degree of valley polarization *ρ*_VP_ for *σ*+ excitation is defined as *ρ*_VP_ = (*I*_*σ*+_ − *I*_*σ*−_)/(*I*_*σ*+_ + *I*_*σ*−_), where *I*_*σ*+_ (*I*_*σ*−_) is the intensity of the collected light with *σ*+ (*σ*−) orientation. Valley coherence measurements are similar except measurements are carried out in a linear basis with co-polarized (∥) and cross-polarized (⊥) configurations as shown in Fig. 5c. The degree of valley coherence *ρ*_VC_ is calculated similarly as *ρ*_VC_ = (*I*_∥_ + *I*_⊥_), where *I*_∥_ (*I*_⊥_) is the intensity of the collected light that is ∥(⊥) to the incident light.

Alloy-dependent values of *ρ*VP for X^0^ (black squares) and X^T^ (red circles) at 5 K are plotted in Fig. 5d. We find that *ρ*_VP_ ≈ 49% for X^0^ in WSe_2_, which agrees with previously reported values^9^. Achieving this value required cleaning our heterostructures postassembly using the nano-squeegeeing technique^31^. This process improved the WSe_2_ *ρ*_VP_ of X^0^ by a factor of ≈1.7, thus increasing the signal from 29% to 49%, whereas it appeared to have very little effect on *ρ*_VC_. This result illustrates the impact of contaminants on valley properties and a comparison of squeegeed and non-squeegeed alloys can be found in Supplementary Fig. 9.

As Te is substituted into the lattice, *ρ*_VP_ first remains unchanged at *x* = 0.04 and then decreases to 32% at *x* = 0.14 (Fig. 5d). For *x* > 0.14, no valley polarization is observed. X^T^ exhibits a similar trend with *x*: *ρ*_VP_ ≈ 67% for X^T^ in WSe_2_ and is zero for *x* > 0.14. To the best of our knowledge, there is no systematic theory for understanding valley depolarization from alloy disorder. However, we do observe an interesting correlation between *ρ*_VP_ and the integrated intensity ratio of X^T^/X^0^, as shown in Supplementary Fig. 10. The *ρ*_VP_ X^T^/X^0^ integrated intensity ratio generally decreases with Te incorporation from ≈2.4 in WSe_2_ to ≈0.25 in *x* = 0.37. While there is an initial increase in the *ρ*_VP_ X^T^/X^0^ intensity ratio from ≈2.4 in WSe_2_ to ≈3.8 in the *x* = 0.04 sample, this is most likely the result of a superior cleaned interface via the nano-squeegee method rather than an intrinsic material property. The decrease in the *ρ*_VP_ X^T^/X^0^ intensity ratio as *x* is increased suggests a connection between *ρ*_VP_ and an apparent reduction in doping with increasing Te substitution. Valley coherence is only present for neutral excitons^9^ and we plot *ρ*_VC_ versus *x* at 5 K in Fig. 5e. *ρ*_VC_ ≈ 20% in WSe_2_, which is slightly lower than the literature value^9^, and decreases to ≈13% when *x* = 0.04 after which point it remains essentially constant until the 1H-1T_d_ phase transition. We note that valley coherence remains finite even when valley polarization goes to zero at large *x*.

**Fig. 6:**
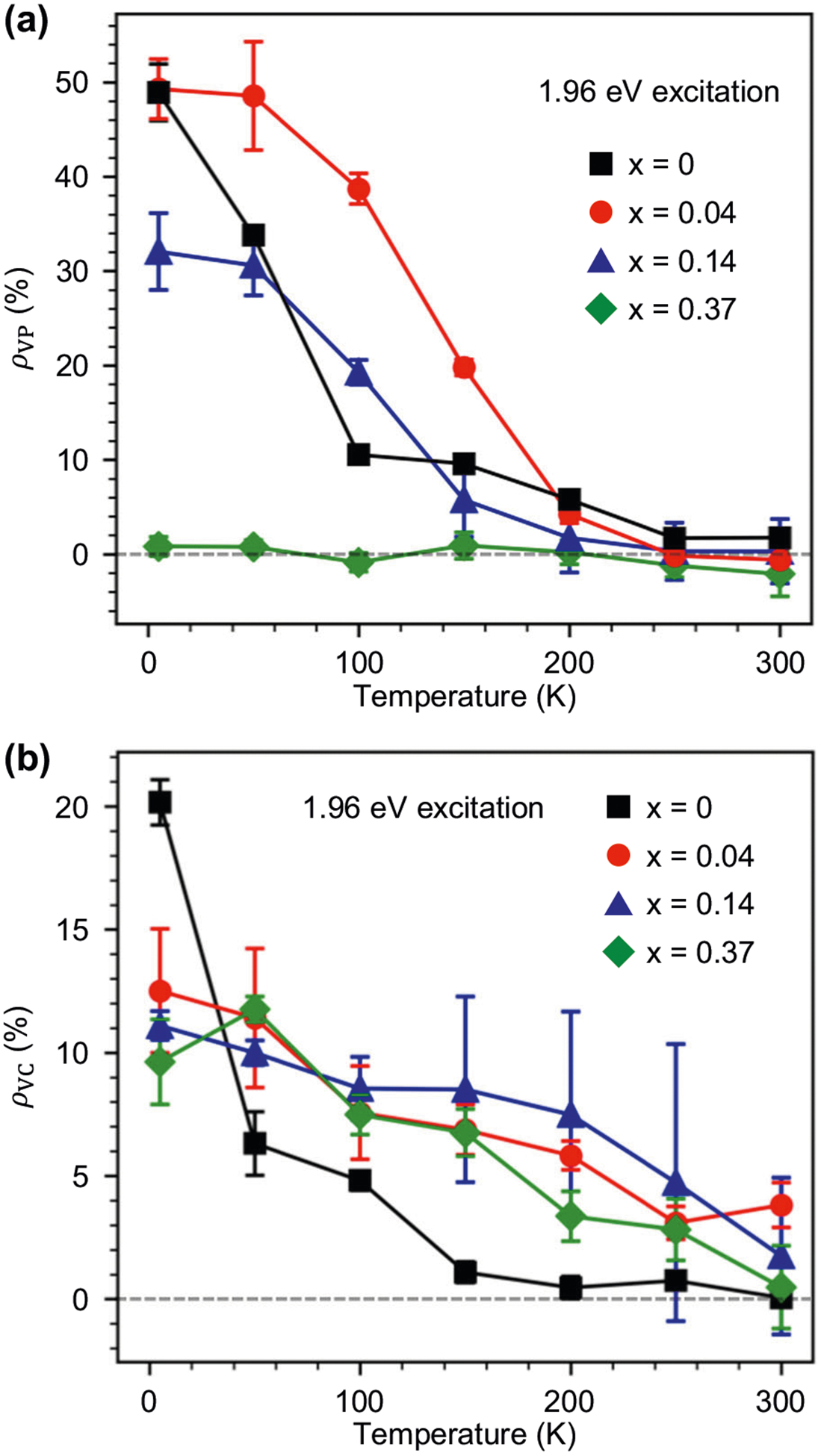
Temperature dependence of the degree of valley polarization (*ρ*_VP_) and the degree of valley coherence (*ρ*_VC_) in 1H-WSe_2(1−x)_Te_2x_. **a** *ρ*_VP_ and **b** *ρ*_VC_ of 1H-WSe_2(1−*x*)_Te_2*x*_ as a function of temperature. In both cases, the alloys are found to sustain valley properties at elevated temperatures when compared to pure WSe_2_ (*x* = 0). Measurements in both panels are done with 1.96 eV excitation. The solid lines are guides to the eye and the error bars are equal to one standard deviation.

Temperature-dependent measurements of *ρ*_VP_ and *ρ*_VC_ for X^0^ in Fig. 6 show interesting behaviors that suggest disorder can in some cases improve valley polarization and valley coherence. In all cases for the 1H-phase alloys, we find a decrease of both quantities with increasing temperature. However, alloys exhibit a different temperature dependence when compared to WSe_2_ (*x* = 0). Beginning with *ρ*_VP_ in Fig. 6a, we observe a sharp decrease with increasing temperature that is consistent with prior examinations of WSe_2_ monolayers^12,60^. Surprisingly, we find that valley polarization remains large for the *x* = 0.04 alloy and can exceed that of WSe_2_, reaching a maximum enhancement factor of 3.5× at 100 K. While valley polarization is overall lower for the *x* = 0.14 alloy, it also exceeds that of WSe_2_ at 100 K and has the same temperature dependence as the *x* = 0.04 case. We note that *ρ*_VP_ of X^T^ also shows similar temperature and alloy dependence, which can be seen in Supplementary Fig. 11. This observation indicates that, counter to intuition, alloys can exhibit valley polarization that meets or exceeds that of WSe_2_, especially at higher temperatures.

Currently, there is no systematic understanding of the impact of alloy disorder on valley polarization. The prevailing theories of valley polarization revolve around a balance between the mean exciton lifetime *τ*_x_ and the valley relaxation lifetime *τ*_*v*_, which are parametrized by the relationship $\rho_{\text{VP}}\;=\;\frac{\rho_{0}}{1+2\left(\gamma_{\text{v}}/\gamma_{\text{x}}\right)}$, where *γ*_v =_ (2*τ*_v_)^−1^ is the intervalley scattering rate and *γ*_x =_ (*τ*_x_)^−1^ is the exciton recombination rate^8,19^. Temperature dependence enters this equation via the scattering and recombination rates, and the valley depolarization rate for excitons is determined by a combination of electron–hole exchange interactions^8,17^, intervalley phonon scattering^7,18^, and Coulomb screening^61^ of the exchange interaction. Miyauchi et al.^19^ more deeply explore the temperature dependence of *τ*_v_ and find that valley depolarization at low temperatures is driven by long-range electron–hole exchange interactions, but as temperatures are increased, intervalley phonon scattering dominates. A subsequent experimental study demonstrated that the valley polarization can be enhanced over a range of temperatures by electrostatic gating, which adds additional carriers to the material that screen the electron–hole exchange interaction^20^. Therefore, we suggest that carrier doping may be a contributing factor to the enhancement of *ρ*_VP_ in alloys at elevated temperatures, which is supported by the larger X^T^/X^0^ integrated intensity ratio for *x* = 0.04 compared to WSe_2_ (Supplementary Fig. 10). However, we cannot conclude that doping is the only factor since the X^T^/X^0^ integrated intensity ratio of the *x* = 0.14 alloy is less than that of WSe_2_ but its valley polarization is larger at 100 K. Another possible explanation for the sustained valley polarization at higher temperatures may be a reduction in *τ*_x_ due to disorder^62^. We have attempted to fit our data using functional forms provided by Miyauchi et al.^19^ but cannot obtain unique fitting parameters since both *τ*_x_ and *τ*_v_ are complicated functions of temperature, bright-dark exciton splitting, phonon scattering rates, and exciton relaxation times.

The valley decoherence rate differs from the valley depolarization rate by its additional sensitivity to pure dephasing (*γ*_dep_). This results in a different expression $\rho_{\text{VC}}\;=\;\frac{\rho_{0}}{1+2\left(\gamma_{\text{v}}+\gamma_{\text{dep}}\right)/\gamma_{\text{x}}}$^63^, ^64^. Unique temperature dependencies for *ρ*_VP_ and *ρ*_VC_ are therefore expected and observed in Fig. 6a and Fig. 6b, respectively. We again find that for alloys the valley coherence is larger than in WSe_2_ at higher temperatures. The impact of pure dephasing on valley coherence makes it more sensitive to scattering events than valley polarization. According to Hao et al.^11^, changes in exciton momentum due to scattering from defects yields an in-plane magnetic field that leads to depolarization and decoherence. As the frequency of impurity scattering is increased, however, the time-averaged effective magnetic field experienced by excitons due to the electron–hole exchange interaction^17^ is reduced, which can act to enhance valley properties. Further studies on WSe_2(1−*x*)_Te_2*x*_ using temperature-dependent and time-resolved spectroscopies such as those conducted by Miyauchi et al.^19^ and Hao et al.^11^ are required to determine the respective contributions of the above decoherence and depolarization mechanisms.

## Discussion

We have used low-temperature Raman and temperature-dependent, polarization-resolved PL spectroscopy to characterize different crystal phases spanned by monolayer WSe_2(1−*x*)_Te_2*x*_ alloys and explore how incorporation of Te into the WSe_2_ lattice affects valleytronic and semiconducting properties. DFT calculations of the phonon dispersion curves for 1H-WSe_2_ and 1T_d_-WTe_2_ alongside low-temperature Raman measurements allowed us to assign the vibrational modes of the WSe_2_ and WTe_2_ endpoint compounds. The shifting and splitting of these vibrational modes were tracked with composition *x*, and we found the appearance of alloy-only features resulting from W-Te vibrations in the 1H alloys that we confirm through the combination of polarization-resolved Raman measurements and DFT calculations. Temperature-dependent PL measurements were used to demonstrate band gap tunability, identify the alloy dependence of exciton and trion states, and observe a new defect-related emission feature. DFT calculations of the optical band gap in the alloys agree very well with low-temperature PL measurements when using the HSE06 functional. Polarization-resolved PL measurements show that alloys still exhibit valley polarization and coherence and that these valley properties can be superior to those of WSe_2_ at higher temperatures. Reflectance measurements were also used to measure the A and B excitons in select alloys, indicating that the spin–orbit splitting of the valence band can be increased with the addition of Te. This study illustrates the resilience of valley phenomena in alloys and the prospect of their application in a novel class of phase change memory technologies that also take advantage of spintronic and valleytronic information processing.

## Methods

### Crystal growth and structural characterization.

WSe_2(1−*x*)_Te_2*x*_ alloys (*x* = 0…1) were grown by the chemical vapor transport method. Appropriate amounts of W(99.9%), Se (99.99%), and Te (99.9%) powders were loaded in quartz ampoules together with ≈90 mg (≈4 mg/cm^3^ of the ampoule’s volume) of TeCl_4_ which served as a transport agent. The ampoules were then sealed under vacuum and slowly heated in a single-zone furnace until the temperature at the source and the growth zones reached 980 and 830 °C, respectively. After 4 days of growth, the ampoules were ice-water quenched. Crystal phases of the alloys were determined by examining powder X-ray diffraction patterns using the MDI-JADE 6.5 software package. We found that alloys with *x* ≤ 0.4 crystallized in the 2H phase and those with *x* ≥0.8 were in the T_d_ phase. Results are consistent with a previous report of WSe_2(1−*x*)_ Te_2*x*_^29^. Chemical compositions were determined by the energy-dispersive X-ray spectroscopy (EDS) using a JEOL JSM-7100F field-emission scanning electron microscope equipped with an Oxford Instruments X-Max 80 EDS detector.

### Sample preparation.

For optical studies, WSe_2(1−*x*)_Te_2*x*_ bulk crystals were mechanically exfoliated and monolayers were identified by optical contrast. Monolayers were then fully encapsulated in an hBN heterostructure (top and bottom layers) using the viscoelastic dry-stamping method on a SiO_2_/Si substrate (90 nm oxide thickness) to protect them from the degradative effects due to exposure to the atmosphere and to provide a uniform dielectric environment^33^. We note that, before encapsulation, monolayers were exposed to the atmosphere for <1 h. To guarantee clean hBN/TMD contact, the nano-squeegee method was used with a scan line density of ≥10 nm/line and a scan speed of ≤30 μm/s as suggested by Rosenberger et al.31 to physically push contaminants out from in between heterostructure interfaces. Results of this procedure can be seen in the atomic force microscopic image of Supplementary Fig. 6. Here the contaminants removed from the interfaces are gathered along both sides of the nano-squeegeed region (dark vertical lines, ≈50 nm in height above sample).

### Optical studies.

Raman and PL measurements were performed on home-built confocal microscopes, both in backscattering geometries, that were integrated with a single close-cycle cryostat (Montana Instruments Corporation, Bozeman, MT). A 532 nm laser was used for Raman measurements since it has been shown that this excitation source can excite first- and second-order features^34^, whereas a 633 nm laser was used for PL measurements since it has been shown to yield a much higher degree of valley polarization than excitation with a green laser^16^. Both set-ups focus the excitation source through a 0.42 NA long working distance objective with ×50 magnification. For Raman measurements, the laser spot was ≈2.4 μm, and the laser power density was fixed at 66 μW/μm^2^, while for PL measurements, the laser spot was determined to be ≈2.2 μm, and the laser power density was fixed at 21 μW/μm^2^. Collected light in both cases was directed to a 500 nm focal length spectrometer with a liquid nitrogen-cooled CCD (Princeton Instruments, Trenton, NJ). The spectrometer and camera were calibrated using a Hg-Ar atomic line source. For spectral analysis, Raman peaks were fit with Lorentzian profiles, whereas PL peaks were fit with Gaussians.

### DFT calculations.

DFT calculations were performed using the Vienna ab initio Simulation Package^65–67^. Projector augmented wave pseudopotentials^68^ and the PBE exchange-correlation functional^55^ were utilized. Spin–orbit coupling was included in all calculations except for the phonon band structure, which is a standard procedure^69^. It has been recently reported that there are slight differences between the 1T’ and 1T_d_ phases in monolayer TMDs^32^, with WTe_2_ likely forming in the 1T_d_ phase. However, in this work we performed all calculations with the WTe_2_ monolayer in the 1T’ structure. Owing to very small differences between the 1T’ and 1T_d_ phases, this assumption is appropriate. Full relaxations of the lattice parameters and ionic positions were performed on monolayer WSe_2_ and WTe_2_ in the 1H and 1T’ phases using a 32 × 32 × 1 and 32 × 16 × 1 *Γ*-centered *k*-mesh, respectively, and a 500 eV plane-wave cutoff. The phonon band structures of these compounds were computed using density functional perturbation theory^70^. Various chalcogen-alloyed compositions of the form WSe_2(1−*x*)_Te_2*x*_ were created by expanding these unit cells and substituting the appropriate amount of Te with Se (or vice versa). Full relaxations were again performed in each case, with the *k*-mesh scaled appropriately to the size of the unit cell. In the case when multiple substitutional anions were needed to achieve a given composition, all combinations of the position of the alloying atoms relative to each other were tested, with the lowest energy configuration considered the ground state (Supplementary Fig. 12). The density of states was computed using the aforementioned PBE functional, as well as the HSE06 functional56, with 25% Hartree–Fock exact exchange included.

## Supplementary Material

1

## Figures and Tables

**Table 1:** Raman mode assignments.

1H-WSe_2_		
Label	Position (cm^−1^)	Assignment
1	132	Unknown
2	223	*E*′ (*K*)^a^
3	240	*E′* (*M*)^a^
4	250	$A_{1}^{\prime }$ + *E*′
5	264	2*LA*(*M*)^a,b^
6	351	2*E*′(Γ)^b^ or $A_{1}^{\prime }$ (*M*) + *TA*(*M*)^b^
7	378	[*E*′(Γ) *or* $A_{1}^{\prime }$ (Γ)] + *LA*(*M*)^a,b^
8	401	[*E*′(Γ) *or* $A_{1}^{\prime }$ (Γ)] + *LA*(*K*)^a^ or 3*LA*(*M*)^a,b^
T_d_-WTe_2_ (bilayer)		
Label	Position (cm^−1^)	Assignment
a	87	*A*_2_
b	107	*A*_2_
c	166	*A*_1_
d	218	*A*_1_
e	327	2*A*_1_ (2 × peak c) or *A*_1_+ *A*_2_ (peak d + peak b)

1H-WSe_2_ and T_d_-WTe_2_ (bilayer) vibrational mode symmetry assignments for the peaks identified in Fig. 2a, b. WSe_2_ peaks are labeled with numbers and WTe_2_ peaks are labeled with letters. The superscripts a and b refer to assignments made by del Corro et al.^34^ and Zhaoet al.^35^, respectively
