# Supplementary material for: Valley phenomena in the candidate phase change material WSe2(1−x)Te2x
Source: Commun Phys. Author manuscript; Available in PMC 2020 Nov 19. (PMC7675920)
Supplement: 1 [file NIHMS1588378-supplement-1.pdf]

Supplementary Information

Supplementary Figures

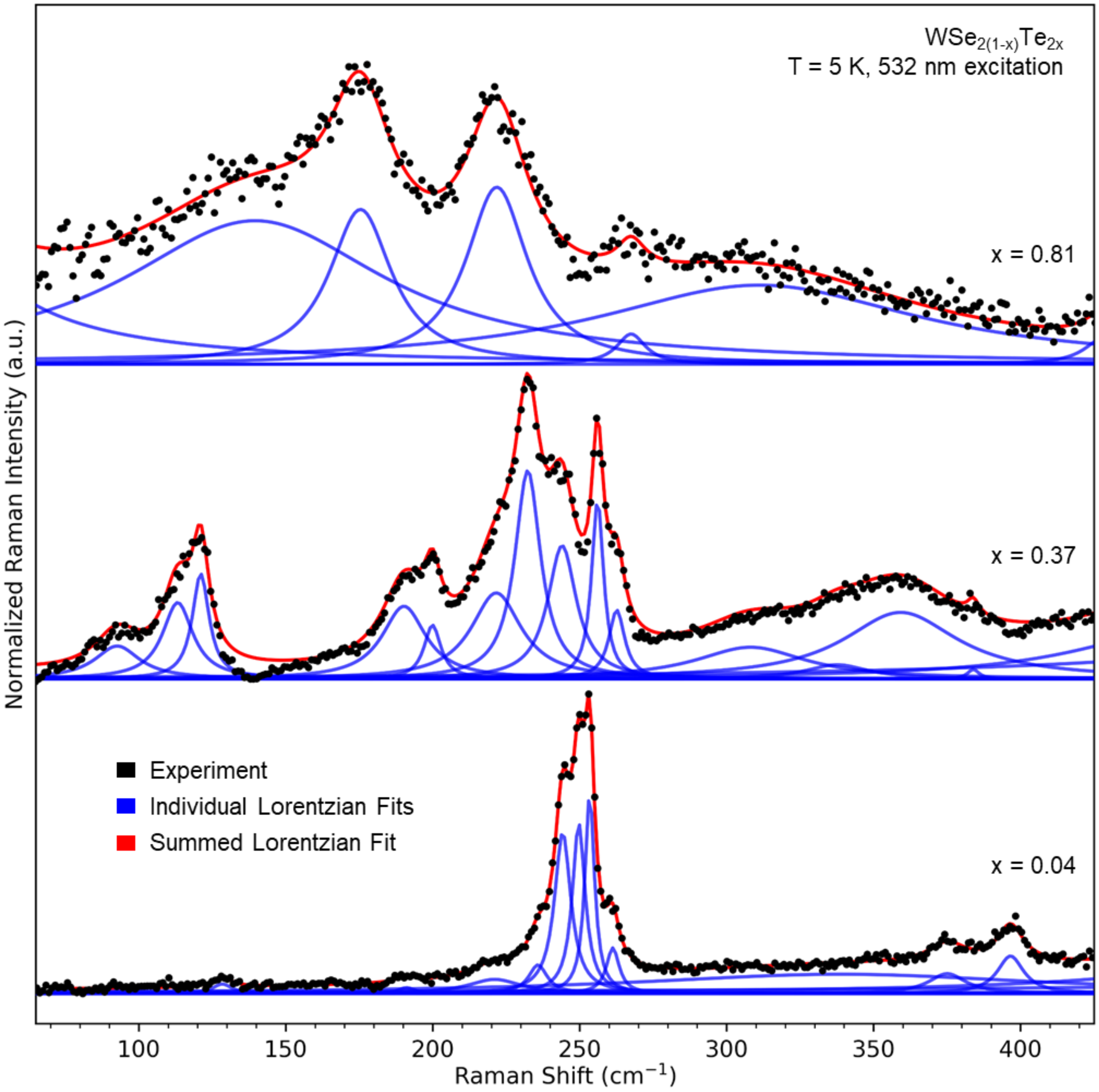

Supplementary Figure 1 | Lorentzian fits to Raman data of select $WSe_{2(1-x)}Te_{2x}$ alloys. Raman measurements of x = 0.04, 0.37, and 0.81 alloys taken at 5 K with 532 nm excitation. The black points correspond to the experimental data and the blue peaks are individual Lorentzian fits. The red curves are obtained by summing the individual Lorentzian fits. The spectra are offset vertically for clarity.

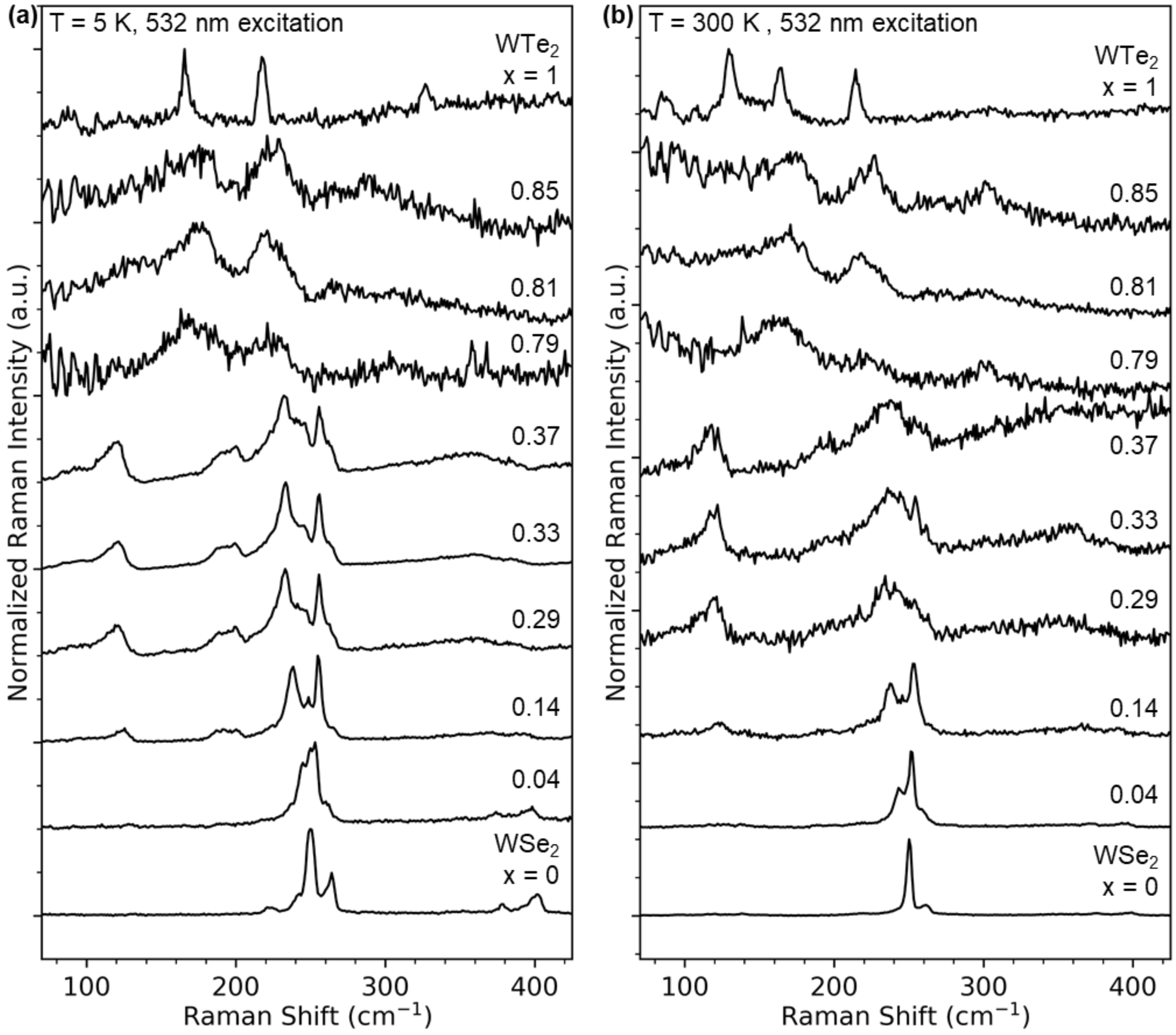

Supplementary Figure 2 | Composition-dependent Raman measurements of $WSe_{2(1-x)}Te_{2x}$. Raman measurements taken with 532 nm excitation at **(a)** 5 K and **(b)** 300 K for all studied compositions. We note that even though the WTe_2 spectra of panels **(a)** and **(b)** were taken on the same sample, the spectrum of panel **(a)** was taken on a bilayer region (absence of peak at ≈130 cm^{-1}) while the spectrum of panel **(b)** captured a nearby portion of bulk WTe_2 (presence of peak at ≈130 cm^{-1}).

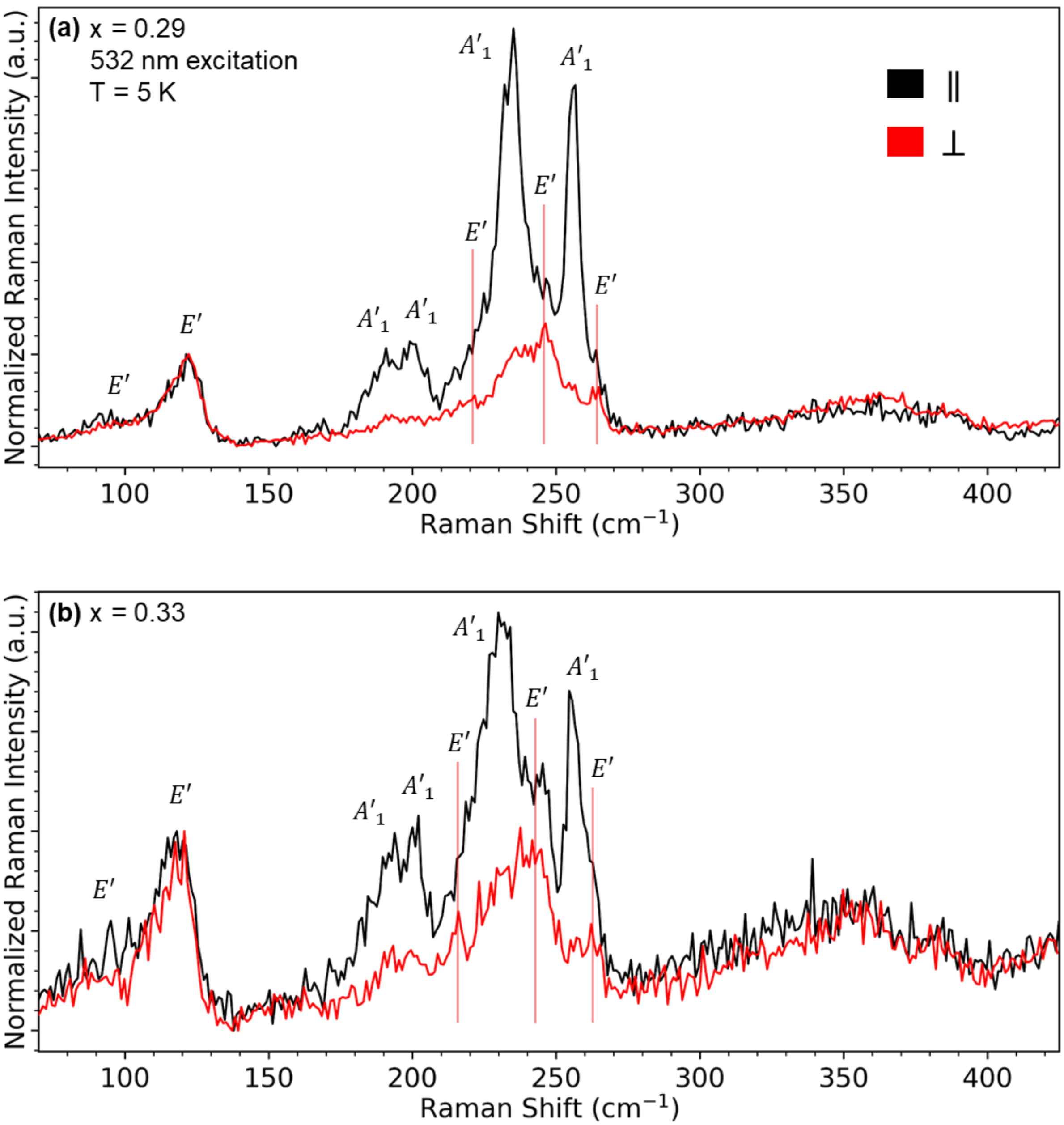

Supplementary Figure 3 | Polarization-resolved Raman measurements of select 1H-$WSe_{2(1-x)}Te_{2x}$ alloys taken at 5 K. Raman measurements taken with 532 nm excitation of **(a)** x = 0.29 and **(b)** x = 0.33 alloys, which were chosen for the clear splitting of the primary A'_1 mode in WSe_2. Due to the differing symmetries of the A'_1 and E' modes, polarization selection rules in a backscattering experimental geometry state that A'_1 modes should only be visible when the analyzer is co-polarized (∥) with the excitation, while E' modes are visible when the analyzer is arranged either ∥ or cross-polarized (⊥) with the excitation.

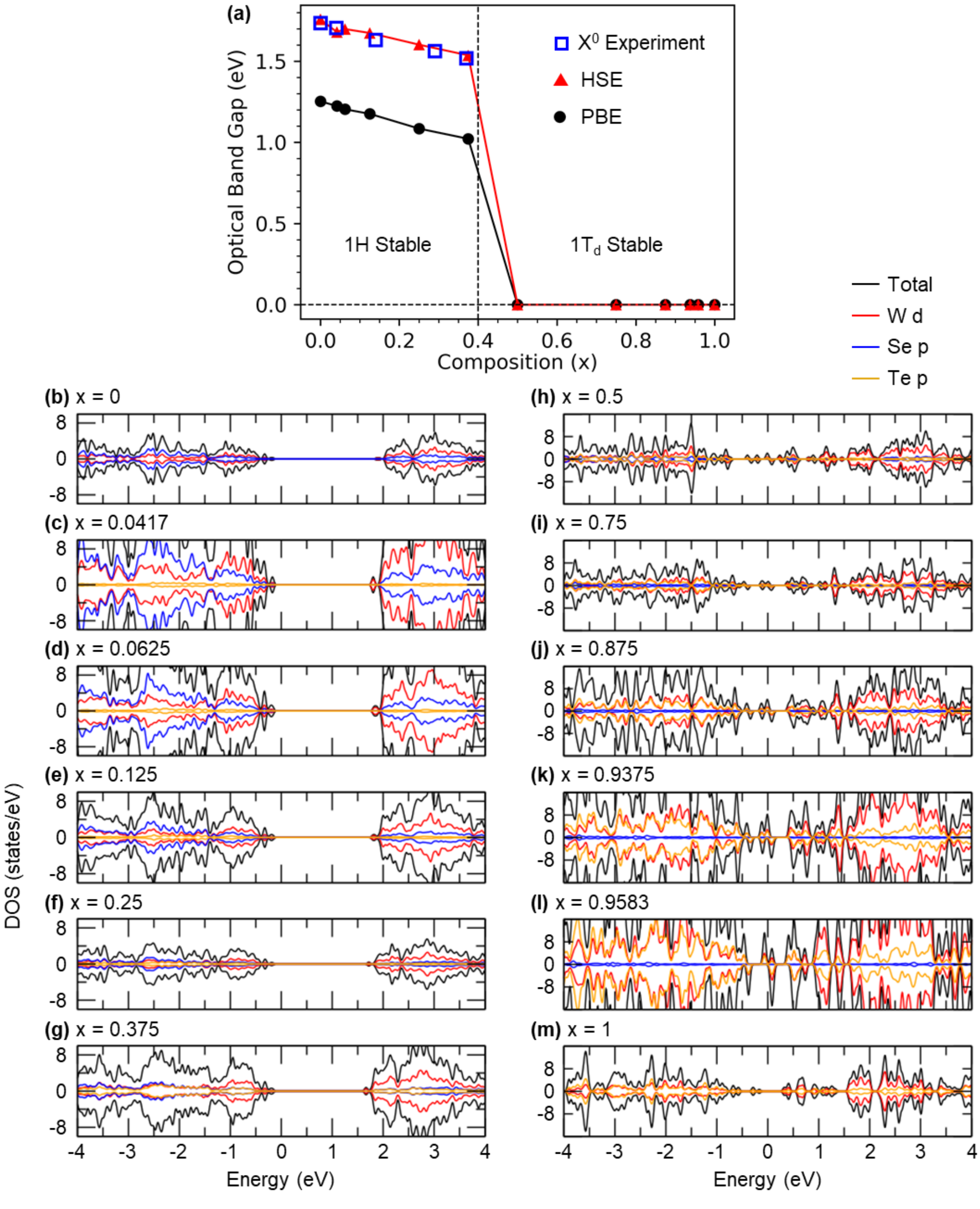

Supplementary Figure 4 | Density functional theory (DFT)-calculated optical band gaps and density of states for $WSe_{2(1-x)}Te_{2x}$. (a) Optical band gaps calculated using DFT. We find that the HSE06 functional predicts a band gap that agrees better with experiment than the PBE functional as demonstrated in several other material families.[1,2] **(b-m)** Density of states calculated using the HSE06 functional for monolayer $WSe_{2(1-x)}Te_{2x}$ alloys.

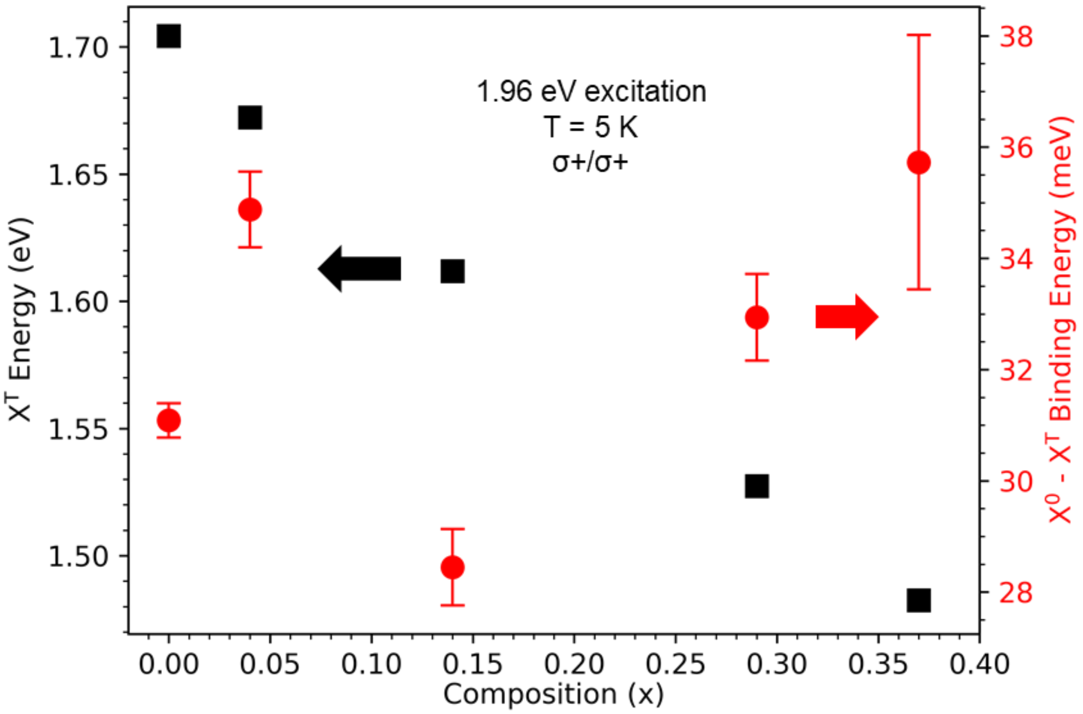

Supplementary Figure 5 | Trion (X^T) energy and neutral exciton (X^0) - X^T binding energy vs. composition x of 1H-$WSe_{2(1-x)}Te_{2x}$ alloys. The alloy dependence of the X^T energy is plotted as black squares (left axis) and the alloy dependence of the X^0 - X^T binding energy is plotted as red circles (right axis). Data was taken at 5 K with 1.96 eV excitation. Excitation and collection were done with right circularly polarized light (σ+). The error bars are equal to one standard deviation and are smaller than the data point for X^T energy.

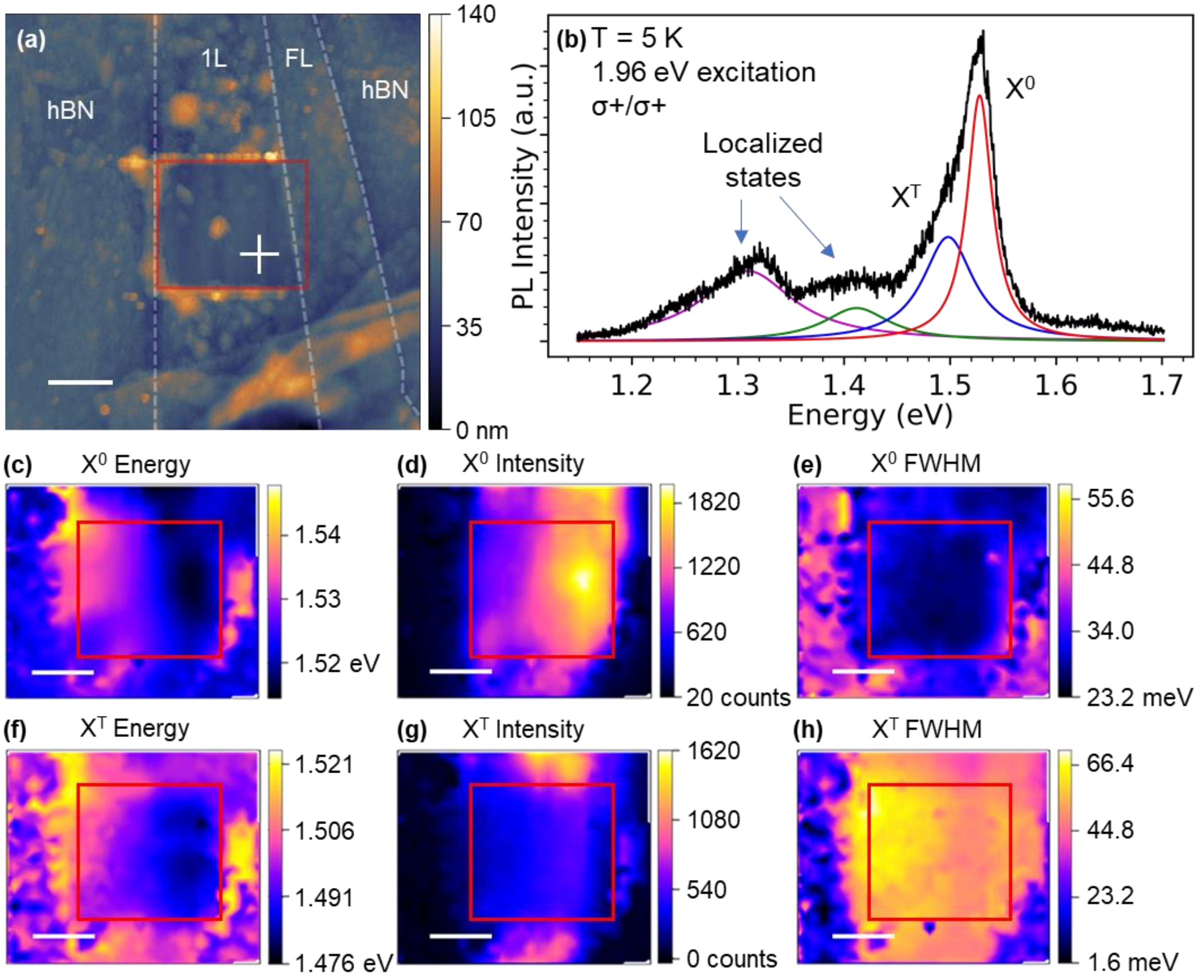

Supplementary Figure 6 | Nano-squeegeeing of $WSe_{2(1-x)}Te_{2x}$ van der Waals heterostructures and photoluminescence (PL) spatial mapping. (a) Atomic force microscope image of a hexagonal boron nitride (hBN)-encapsulated x = 0.33 sample that has been nano-squeegeed (region outlined with a red box) to clean the heterostructure's interfaces. Monolayer (1L) and few-layer (FL) regions have been labeled and outlined with white dashed lines to distinguish from areas with only top and bottom layers of hBN and no transition metal dichalcogenide in between. Residues removed from the heterostructure's interfaces are gathered around the edges of the nano-squeegeed region. **(b)** Low-temperature (5 K) PL spectrum taken with 1.96 eV excitation at the location marked by the white crosshairs in panel **(a)**. The sample is excited with right circularly polarized light σ+ and re-emitted σ+ light is collected. Lorentzian fits to the neutral exciton (X^0), trion (X^T), and localized states are shown. PL spatial mapping of the **(c)** X^0 energy, **(d)** intensity, and **(e)** full width at half maximum (FWHM), as well as the **(f)** X^T energy, **(g)** intensity, and **(h)** FWHM. The nano-squeegeed region is outlined with a red box in panels **(c)** - **(h)** as well. All scale bars are 3 μm.

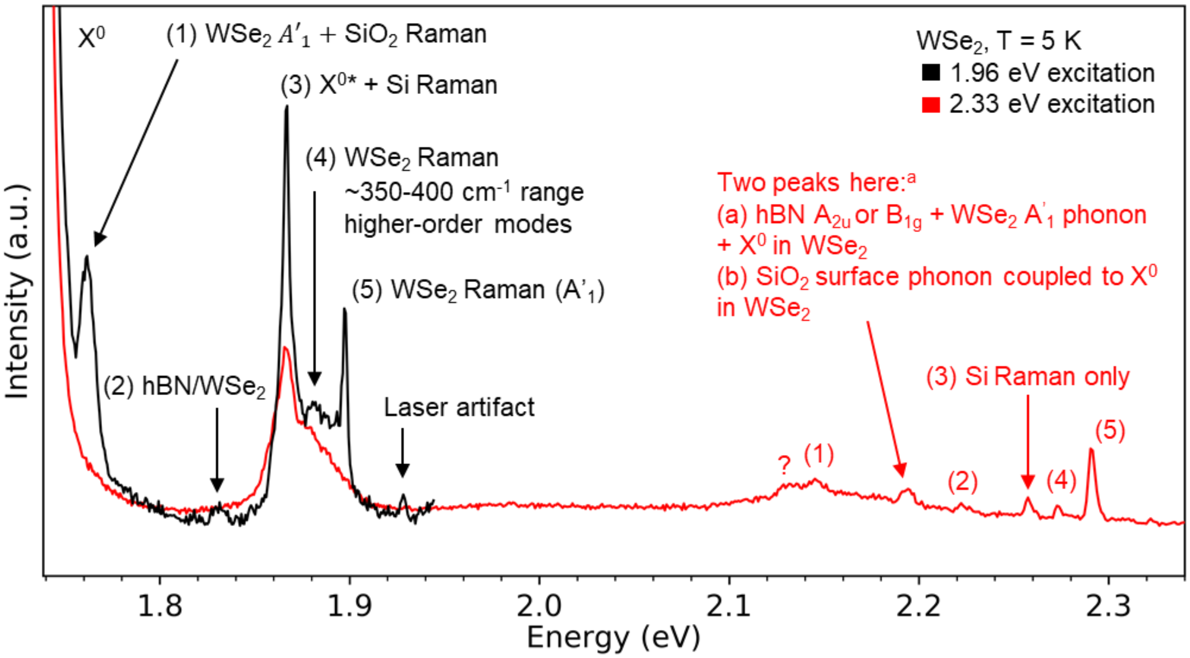

Supplementary Figure 7 | Zoom-in of exciton and exciton-phonon complexes in WSe_2 at energies above the neutral exciton (X^0) that occur at 5 K. Here, measurements are done with 1.96 eV excitation (black curve) and 2.33 nm excitation (red curve) to determine whether the features are related to optical transitions or Raman scattering. Peaks common to both measurements are labeled with numbers (1) - (5). The peak (3) X^{0*} feature originating from the *2s* state of the A exciton does not shift with excitation energy, although it appears to decrease in intensity with 2.33 eV excitation since it no longer overlaps with the Si Raman peak as it does under 1.96 eV excitation. The peaks (1) - (5) that are related to Raman scattering shift in energy by the same amount as the change in excitation energy. The black curve ends just above 1.92 eV due to the collection cutoff filter. The superscript a refers to assignments made by Chow et al.[5]

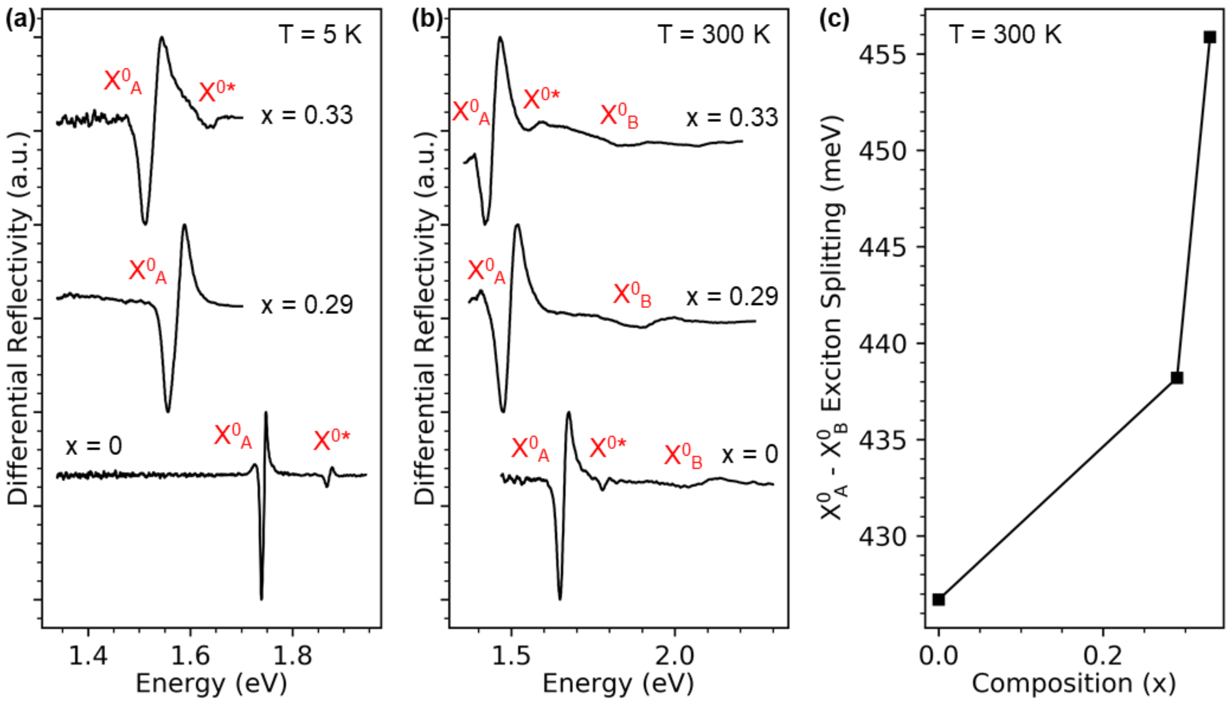

Supplementary Figure 8 | Differential reflectivity measurements of select $WSe_{2(1-x)}Te_{2x}$ alloys. Measurements are taken at **(a)** 5 K and **(b)** 300 K. The A exciton ($X^0{}_A$), B exciton ($X^0{}_B$), and *2s* exciton (X^{0*}) are labeled. Prior to differentiation, reflectivity measurements were processed using the equation $\frac{R_{\mathrm{TMD}}-R_{\mathrm{Substrate}}}{R_{\mathrm{TMD}}-Dark}$, where R_{TMD} and $R_{\mathrm{Substrate}}$ are reflectance measurements taken on the encapsulated transition metal dichalcogenide (TMD) alloy and the top and bottom hBN sandwich atop the SiO_2/Si substrate without the TMD alloy in between, respectively. $Dark$ refers to a dark scan to remove any background effects. Spectra are cleaned using a Savitzky-Golay filter. **(c)** The 300 K splitting between the A and B excitons is estimated from the zero crossings of the data in panel (b) and are plotted against alloy composition x.

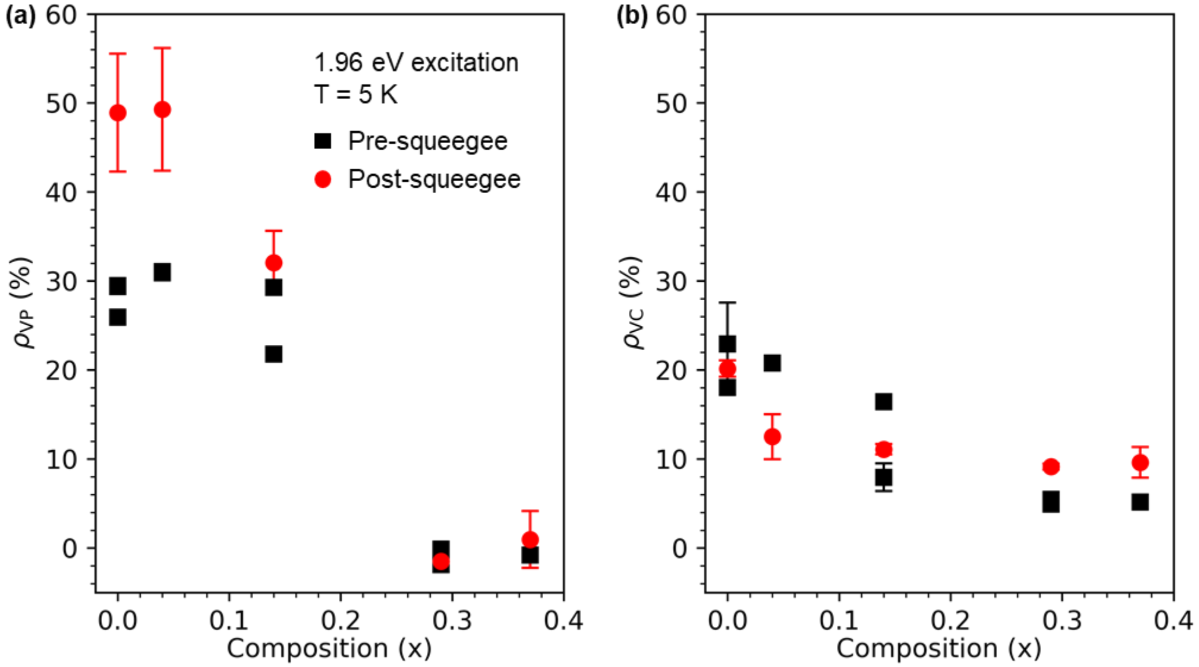

Supplementary Figure 9 | Valley phenomena in 1H-$WSe_{2(1-x)}Te_{2x}$ before and after nano-squeegeeing. Comparison of **(a)** the degree of valley polarization (ρ_{VP}) and **(b)** the degree of valley coherence (ρ_{VC}) for the neutral exciton in pre- and post-squeegeed samples (black squares and red circles, respectively). The nano-squeegeeing process improved ρ_{VP}, but had very little effect on ρ_{VC}. The error bars in both panels are equal to one standard deviation.

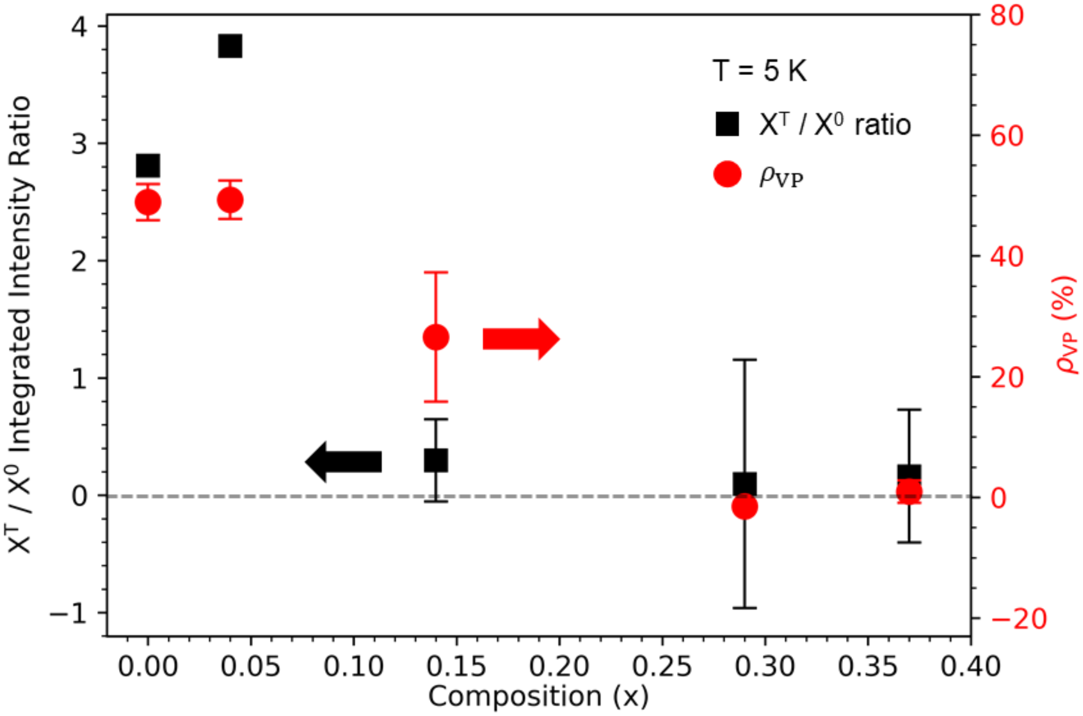

Supplementary Figure 10 | Alloy dependence of the trion (X^T) / neutral exciton (X_0) integrated intensity ratio and the degree of valley polarization (ρ_{VP}) of X^0 in 1H-$WSe_{2(1-x)}Te_{2x}$ alloys at 5 K. The X^T / X^0 ratio values are marked by black squares and correspond to the left axis. The ρ_{VP} values are marked by red circles and correspond to the right axis. Measurements are done with 1.96 eV excitation at 5 K. The error bars are equal to one standard deviation.

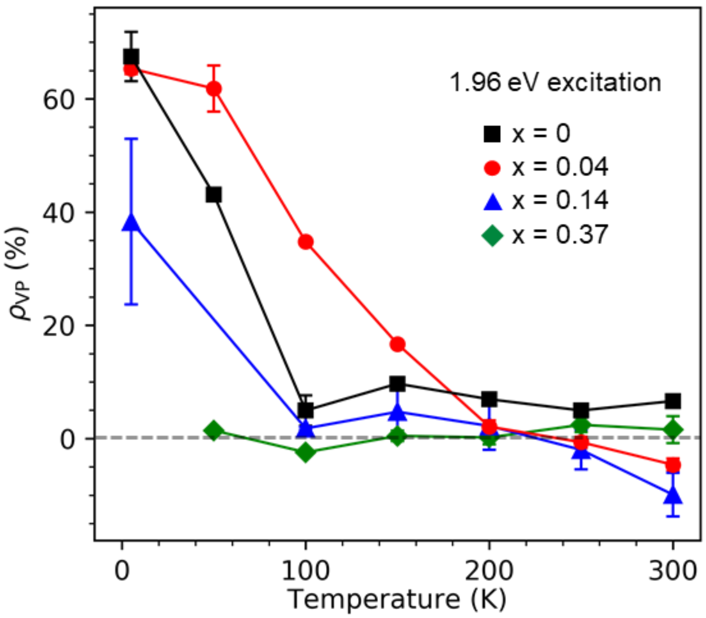

Supplementary Figure 11 | Temperature dependence of the degree of valley polarization (ρ_{VP}) for the trion (X^T) in 1H-$WSe_{2(1-x)}Te_{2x}$ alloys. ρ_{VP} is found to be sustained at higher temperatures for the x = 0.04 sample than for pure WSe_2 (x = 0). Measurements are done with 1.96 eV excitation. The error bars are equal to one standard deviation.

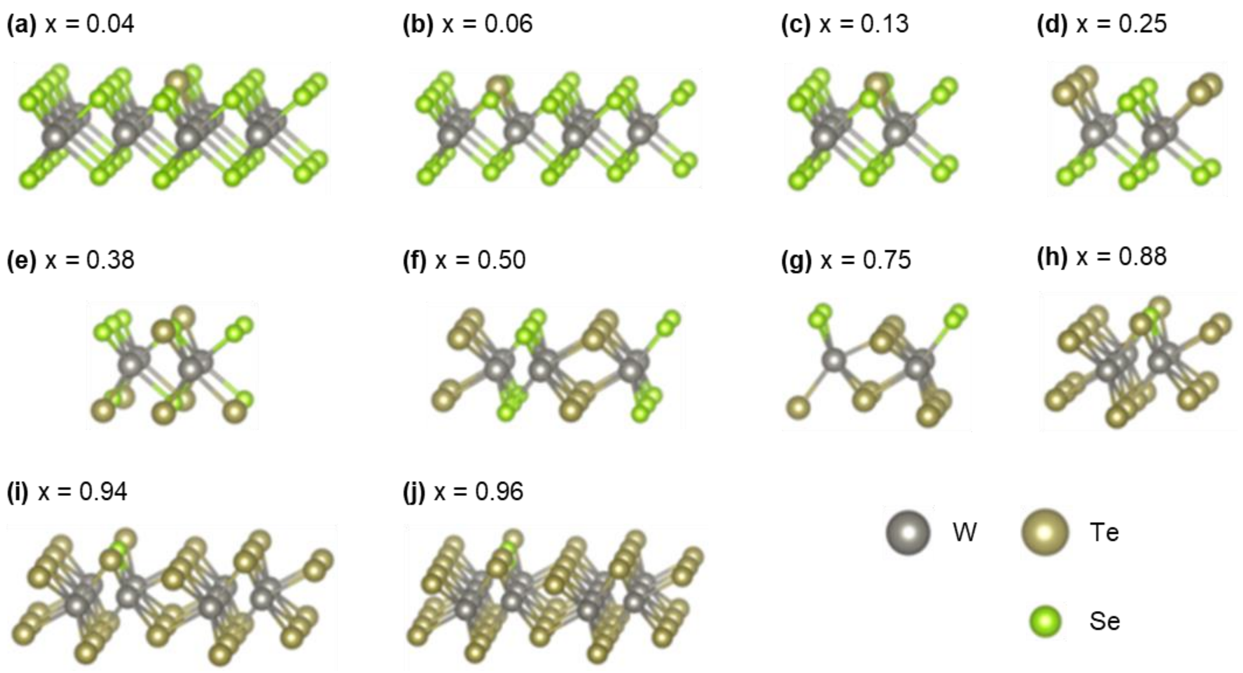

Supplementary Figure 12 | $WSe_{2(1-x)}Te_{2x}$ ground state structures investigated using density functional theory (DFT). The structures explored using DFT correspond to alloy compositions **(a)** x = 0.04, **(b)** x = 0.06, **(c)** x = 0.13, **(d)** x = 0.25, **(e)** x = 0.38, **(f)** x = 0.50, **(g)** x = 0.75, **(h)** x = 0.88, **(i)** x = 0.94, and **(j)** x = 0.96.

Supplementary Notes

Supplementary Note 1

We use photoluminescence (PL) spatial mapping of an x = 0.33 sample to explore spectral changes across the alloy (**Supplementary Fig. S6**). As with all of the other samples used in this study, the sample was mechanically exfoliated and encapsulated in a hexagonal boron nitride (hBN) heterostructure on a SiO_2/Si substrate using the dry-stamping method with a polydimethylsiloxane polymer.[3] An atomic force microscope (AFM) image of the sample can be seen in **Supplementary Fig. S6a**. We have used the nano-squeegee method[4] to ensure that residues from the encapsulation process have been removed from the areas of interest (red box in **Supplementary Fig. S6a**). Looking closely at the AFM image, it can been seen that not only have impurities been primarily pushed to the edge of the nano-squeegeed region, marked by orange colors indicating a higher height profile where residues have gathered, but some impurities faintly remain in the center and

to the left half of cleaned region. A PL measurement taken at 5 K from the location of the white crosshair in **Supplementary Fig. S6a** is pictured in **Supplementary Fig. S6b**. This spectrum shows the typical features of the alloys, specifically the neutral exciton (X^0), the trion (X^T), and localized states, which in this case have been fit with Lorentzians. Spatial resolution of the energies, intensities, and full widths at half maxima (FWHM) of X^0 and X^T at 5 K are shown in **Supplementary Figs. S6c-S6h**. We focus primarily on the nano-squeegeed region outlined with a red box. In this area, these measurements indicate that while the spatial variations of the FWHMs for X^0 and X^T were miniscule, they were sharpest along the right side of the nano-squeegeed region. The areas of sharpest FWHMs hosted the greatest intensities of X^0 and X^T, as well as a uniform redshift of the peaks' energies.

Supplementary Note 2

We also find evidence for excitons and exciton-phonon complexes at energies above the neutral exciton (X^0) that can be clearly seen in **Supplementary Fig. S7**. The lowest energy feature, labeled WSe_2 A'_1 + SiO_2 Raman, occurs at 1.76 eV with 1.96 eV excitation and shifts with laser energy for 2.33 eV excitation. The energy-dependent position of this feature suggests that this peak has a phonon origin, and was determined by a prior photoluminescence excitation study of different configurations of hBN/WSe_2 encapsulations on SiO_2/Si and sapphire substrates to be a combination of the WSe_2 A'_1 Raman mode and a SiO_2 surface phonon.[5] We measure another feature at 1.83 eV with 1.96 eV excitation, labeled hBN/WSe_2, that shifts with laser energy. As evidenced in previous studies, we attribute this feature to exciton-phonon coupling between hBN and WSe_2 that results in activation of the hBN IR-active A_{2u} ZO mode or the hBN Raman- and IR-silent B_{1g} ZO mode.[5–7] On the other hand, the X^0* peak measured at 1.87 eV does not shift with laser energy, although changing excitation energy results in a decrease in its intensity since this feature no longer rides the Si Raman peak background with which it overlaps when excited with 1.96 eV excitation. The presence of this feature in reflectance measurements at 5 K and 300 K indicates that X^0* results from an optical transition (**Supplementary Fig. S8**). Assignment of this feature to the B exciton is ruled out since the X^0-X^0* splitting at 5 K is ≈110 meV, which is much lower than the A-B exciton splitting of ≈400 meV,[8] and also both the X^{0*} peak and the

transition from the B exciton can be seen together in 300 K reflectance measurements (**Supplementary Fig. S8**). We suggest the X^{0*} feature is the *2s* excited state of X^0,[9] in agreement with prior studies of hBN-encapsulated WSe_2 and $WS_{0.6}Se_{1.4}$.[10–13] We note that there is also a higher energy shoulder accompanying X^{0*} that is independent of laser energy and is thought to be related to the *3s* excited state of X^0.[13] The X^{0*} feature is also observed in 300 K reflectance measurements of the x = 0.33 alloy (**Supplementary Fig. S8**). In the alloy, X^{0*} is ≈120 meV above X^0, suggesting only a small increase in the *1s - 2s* energy splitting with Te incorporation. Reflectance data illustrating the alloy dependence of the A-B exciton splitting at 300 K is also presented in **Supplementary Fig. S8**. This valence band spin-orbit splitting increases with Te composition as expected from prior DFT calculations.[14]

Supplementary References

1. Brothers, E. N., Izmaylov, A. F., Normand, J. O., Barone, V. & Scuseria, G. E. Accurate solid-state band gaps via screened hybrid electronic structure calculations. *J. Chem. Phys.* **129**, 011102 (2008).

2. Ramasubramaniam, A. Large excitonic effects in monolayers of molybdenum and tungsten dichalcogenides. *Phys. Rev. B* **86**, 115409 (2012).

3. Castellanos-Gomez, A. *et al.* Deterministic transfer of two-dimensional materials by all-dry viscoelastic stamping. *2D Mater.* **1**, 011002 (2014).

4. Rosenberger, M. R. *et al.* Nano-"Squeegee" for the Creation of Clean 2D Material Interfaces. *ACS Appl. Mater. Interfaces* **10**, 10379–10387 (2018).

5. Chow, C. M. *et al.* Unusual Exciton–Phonon Interactions at van der Waals Engineered Interfaces. *Nano Lett.* **17**, 1194–1199 (2017).

6. Jin, C. *et al.* Interlayer electron–phonon coupling in WSe_2/hBN heterostructures. *Nat. Phys.* **13**, 127–131 (2017).

7. Du, L. *et al.* Strongly enhanced exciton-phonon coupling in two-dimensional WSe_2. *Phys. Rev. B* **97**, 235145 (2018).

8. Arora, A. *et al.* Excitonic resonances in thin films of WSe_2 : from monolayer to bulk material. *Nanoscale* **7**, 10421–10429 (2015).

9. Wang, G. *et al.* Giant Enhancement of the Optical Second-Harmonic Emission of WSe_2 Monolayers by Laser Excitation at Exciton Resonances. *Phys. Rev. Lett.* **114**, 1–6 (2015).

10. Courtade, E. *et al.* Charged excitons in monolayer WSe_2: Experiment and theory. *Phys. Rev. B* **96**, 1–12 (2017).

11. Meng, Y. *et al.* Excitonic Complexes and Emerging Interlayer Electron–Phonon Coupling in BN

Encapsulated Monolayer Semiconductor Alloy: $WS_{0.6}Se_{1.4}$. *Nano Lett.* **19**, 299–307 (2019).

12. Chen, S.-Y. *et al.* Superior Valley Polarization and Coherence of 2s Excitons in Monolayer WSe_2. *Phys. Rev. Lett.* **120**, 046402 (2018).

13. Manca, M. *et al.* Enabling valley selective exciton scattering in monolayer WSe_2 through upconversion. *Nat. Commun.* **8**, 14927 (2017).

14. Kang, J., Tongay, S., Zhou, J., Li, J. & Wu, J. Band offsets and heterostructures of two-dimensional semiconductors. *Appl. Phys. Lett.* **102**, 012111 (2013).
